# Modulation of metabolic and immunoregulatory pathways in the gut transcriptome of Atlantic salmon (*Salmo salar* L.) after early nutritional programming during first feeding with plant-based diet

**DOI:** 10.3389/fimmu.2024.1412821

**Published:** 2024-07-02

**Authors:** Marwa Mamdouh Tawfik, Mónica B. Betancor, Stuart McMillan, Fernando Norambuena, Douglas R. Tocher, Alex Douglas, Samuel A. M. Martin

**Affiliations:** ^1^ Scottish Fish Immunology Research Centre, School of Biological Sciences, University of Aberdeen, Aberdeen, United Kingdom; ^2^ Hydrobiology Department, Veterinary Research Institute, National Research Centre, Giza, Egypt; ^3^ Institute of Aquaculture, Faculty of Natural Sciences, University of Stirling, Stirling, United Kingdom; ^4^ BioMar AS, Trondheim, Norway; ^5^ Guangdong Provincial Key Laboratory of Marine Biotechnology, Shantou University, Shantou, Guangdong, China

**Keywords:** metabolic programming, first feeding, transcriptome, distal, midgut, mucosal immunity, epigenetic programming, hindgut

## Abstract

**Introduction:**

Plant-based nutritional programming is the concept of exposing fish at very early life stages to a plant-based diet for a short duration to improve physiological responses when exposed to a similar plant-rich diet at a later developmental stage. The mechanisms of action underlying nutritional programming have not been fully deciphered, and the responses may be controlled at multiple levels.

**Methods:**

This 22-week study examines gut transcriptional changes after nutritional programming. Triplicate groups of Atlantic salmon were fed with a plant (V) vs. a marine-rich (M, control) diet for 2 weeks (stimulus phase) at the first exogenous feeding. Both stimulus fish groups (M and V fish) were then fed the M diet for 12 weeks (intermediate phase) and lastly fed the V diet (challenge phase) for 6 weeks, generating two dietary regimes (MMV and VMV) across phases. This study used a whole-transcriptome approach to analyse the effects of the V diet at the end of stimulus (short-term effects) and 22 weeks post-first feeding (long-term effects). After the stimulus, due to its developmental stage, the whole intestine was used, whereas, after the challenge, pyloric caeca and middle and distal intestines were examined.

**Results and discussion:**

At the stimulus end, genes with increased expression in V fish enriched pathways including regulatory epigenetic responses and lipid metabolism, and genes involved in innate immune response were downregulated. In the middle intestine at the end of the challenge, expression levels of genes of lipid, carbohydrate, and energy metabolism were increased in V fish, while M fish revealed increased expression of genes associated with autoimmune and acute adaptive immune response. The distal intestine of V fish showed increased expression of genes associated with immune response and potential immune tolerance. Conversely, the distal intestine of M fish at challenge revealed upregulation of lipid and carbohydrate metabolic pathways, tissue degeneration, and apoptotic responses. The present study demonstrated nutritional programming-associated changes in the intestinal transcriptome, with altered expression of genes involved in both immune responses and different metabolic processes. While there were limited changes in growth between the groups, the results show that there were transcriptional differences, suggesting a programming response, although the mechanism of this response still requires to be fully elucidated.

## 1 Introduction

Due to the finite and limited availability of marine-sourced components, particularly fishmeal and fish oil, their levels must be reduced and replaced with alternatives such as plant-based ingredients to ensure the sustainability of aquaculture practices. Substituting fishmeal and fish oil with plant-based ingredients such as soybean protein concentrate, wheat products, pea protein concentrate, and vegetable oils, including rapeseed oil, has been studied extensively in recent years (1–9). Total replacement has been investigated, revealing some adverse effects on growth and intestinal health (10–13). One reason is that feed intake and nutrient utilisation of plant-based feeds (which requires precise formulation) are impacted by the presence of compounds known as antinutritional factors (e.g., saponins, phytic acid, and proteinase inhibitors) (14). Moreover, plant-based diets lack long-chain polyunsaturated fatty acids (LC-PUFA), particularly the omega-3 LC-PUFA essential for fish and humans (15).

Nutritional programming (NP) as a concept has been investigated in mammals (16–18) and, more recently, in fish (16). In fish, the animals are stimulated with a diet (plant-based, for example) at plastic developmental stages (e.g., first feeding) to induce adaptable physiological changes later in life. Increased growth, feed intake, nutrient digestibility, and efficiency in addition to improved intestinal lining (increased villus length to width ratio) were reported as gross adaptable changes in salmonids and zebrafish (*Danio rerio*) subjected to NP (19–23), but the underpinning mechanisms are unknown. At a hormonal level, appetite-controlling hormones (ghrelin, cholecystokinin, and neuropeptide Y) in zebrafish were changed significantly after NP with plant proteins compared to non-programmed fish, possibly suggesting improved utilisation of plant diet (23). Additionally, lipid metabolism in progeny was affected by rainbow trout (*Oncorhynchus mykiss*) broodstock fed a plant-based diet, suggesting metabolic parental programming with a plant-based diet (24). However, few studies have investigated the metabolic and molecular (gene expression) changes induced by NP (23–25), particularly in the gut.

Further studies deciphered possible epigenetic mechanisms underlying NP in early developmental stages or transgenerational (26–31). Early NP with leucine showed methylation of genes in the mTOR pathway, which is suggested, along with protein synthesis expressed genes, to contribute to improved growth in zebrafish (30); however, epigenetic mechanisms are mainly studied in trans/multigenerational studies known as parental programming. Parent/broodstock fed with α-linolenic acid (ALA) shaped the *scd1a* gene expression in the gilthead sea bream (*Sparus aurata* L.) offspring where those offspring of parents fed the ALA-rich diet revealed an increased DNA methylation (31). Furthermore, parental methionine stimulus impacted DNA methylation of the CpG site of *bnip3a*, among other sites, in the progeny (fry stage) of rainbow trout (29). However, early stimulus by soybean meal showed no lasting (i.e., programming) effects on chromatin modification in gilthead seabream (28). Nevertheless, the epigenetic aspect of NP is still in its infancy compared to that of mammals (16).

Some nutrigenomic research has investigated transcriptomic responses to plant-based diets in the liver and brain of salmonids including rainbow trout (32, 33) and Atlantic salmon (*Salmo salar*) (22). Along with increased feed intake, higher specific growth rate, and improved feed utilisation, transcriptomic changes were observed in rainbow trout brains, suggesting the likelihood of flavour and feed preference acquisition (32). Additionally, the liver transcriptome revealed reduced sensitivity to changes in metabolic and stress pathways in programmed fish (32). Furthermore, upregulated hepatic intermediary metabolism pathways, involved in converting dietary nutrients to cellular components, may partly explain improved nutrient utilisation and performance after NP of Atlantic salmon (22). Immunological tolerance to specific dietary components was also proposed as a possible mechanism underlying NP, which may be reflected in the upregulation of hepatic genes related to anti-inflammatory processes, apoptosis, acquired immune leukocyte receptors, and essential immune response regulators in Atlantic salmon (22), albeit further research is required to support this hypothesis. Moreover, the reduced levels of n-3 LC-PUFA in the plant-based diet resulted in the programmed Atlantic salmon to have upregulated expression of genes involved in LC-PUFA biosynthesis compared to fish exposed to a marine-based diet, which had high dietary n-3 LC-PUFA (22).

The present study explored transcriptomic responses of the intestine in Atlantic salmon fed a plant-based (V) diet as a nutritional stimulus at first feeding. Specifically, this study investigated transcriptomic responses of whole intestine in Atlantic salmon after feeding a V diet vs. a marine-based (M) diet for 2 weeks at first feeding (stimulus phase). After this, all fish were fed the M diet for 14 weeks (intermediate phase), followed by all fish being challenged with a V diet for a further 6 weeks (challenge phase), and the transcriptome [RNA sequencing (RNA-seq)] of different intestinal regions was examined and compared between the fish initially fed (during first feeding) V and M diets at end of the stimulus and challenge phases.

## 2 Materials and methods

### 2.1 Feeding trials, diets, and sampling

Before commencing the study, all experimental protocols were subjected to ethical review by the Animal Welfare and Ethical Review Board, University of Stirling (AWERB (18 19) 045 New ASPA). The feeding experiment was conducted in compliance with the Animals Scientific Procedures Act 1986 (Home Office Code of Practice, HMSO, London, January 1997) under project licence P1A618A4B in accordance with EU regulation (EC Directive 86/609/EEC).

**Figure 1:**
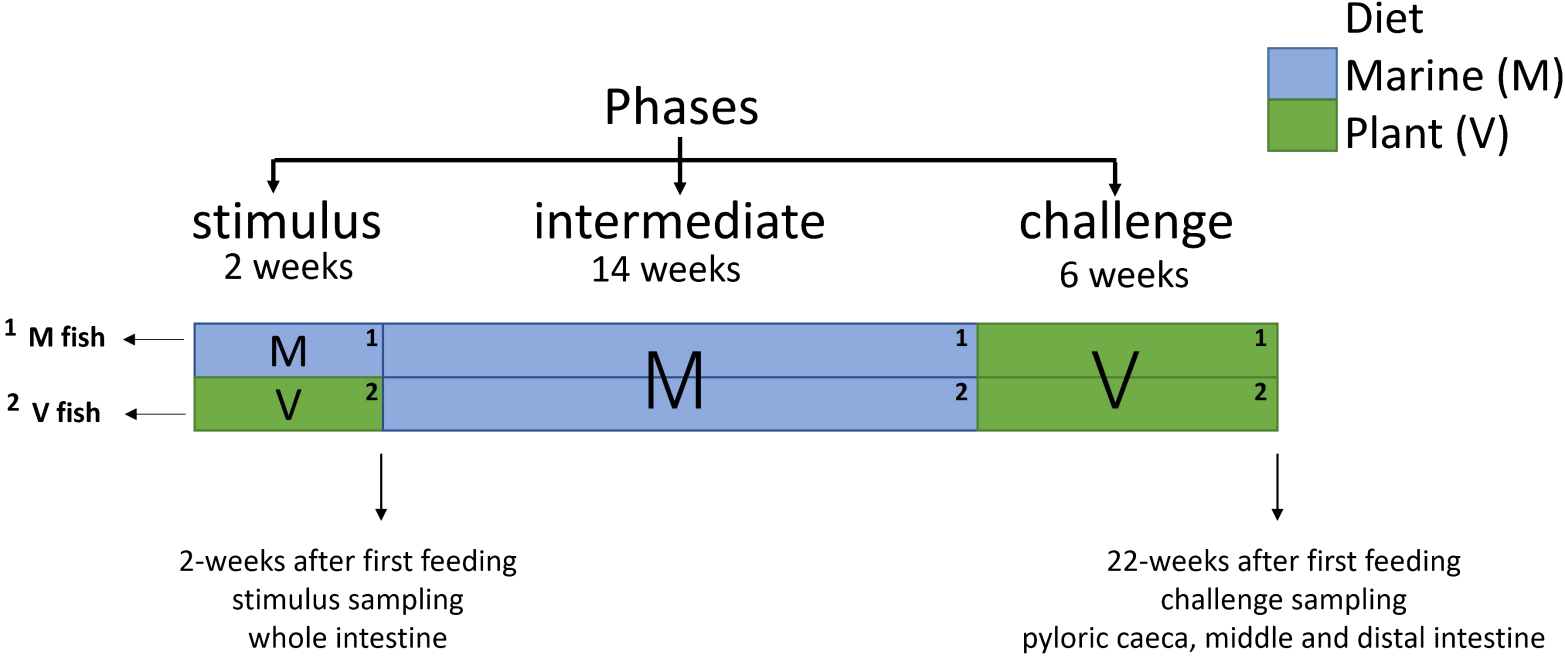
Details of dietary manipulations performed on Atlantic salmon with two sampling points for transcriptome/RNA-seq samples. Gut (whole, plyoric caeca, middle and distal intestines: n = 6 for each gut region) was sampled at the end of phases (sampling points are indicated by arrowheads at the end of the stimulus and challenge phases).

Atlantic salmon (outbreed commercial aquaculture strain, initial weight of 0.152 ± 0.02 g) were kept in triplicate tanks within the RAS system (see water conditions in **Additional file 1**) at the University of Stirling for 22 weeks post-first feeding across three phases comprising a classical NP design (16). At first feeding, fish were fed either an M or V diet for 2 weeks (stimulus phase), followed by all fish being fed an M diet for 14 weeks (intermediate phase), before all fish were transferred to a V diet for a further 6 weeks (challenge phase). Thus, fish were only fed a different diet at first feeding, hence two experimental groups: M or V fish, as all fish were subsequently exposed to the same diets (M diet at the intermediate phase and then V diet at the challenge phase). Thus, fish were fed two dietary regimes (MMV and VMV) in the entire 22-week feeding trial and sampled at the end of the stimulus and challenge phases for short- and long-term effects after NP, respectively. Feeds were adapted based on the fish growth stage including the pellet size (0.5 mm to 2 mm) and protein and lipid contents. Feed formulation, proximate, and fatty acid compositions are provided in McMillan et al. (34) and **Additional file 1**. Briefly, the M diet (stimulus 82% marine meals/4% fish oil) was rich in fishmeal and fish oil, while the V diet (stimulus 5% marine meals/0% fish oil, challenge 10% MM/0% fish oil) contained a combination of soy protein concentrate, corn gluten, and wheat gluten and was supplemented with essential amino acids to meet requirements not provided by the dietary plant ingredients. The fish groups, dietary regimes, and sampling points in the 22-week feeding trial are highlighted in **Figure 1**. Fish were sampled randomly after 24-h starvation and killed with an overdose of anaesthetic (tricaine, 1,000 ppm; MS-222, Pharmaq, Oslo, Norway) followed by a manual cut of the spinal cord. At the end of the stimulus, two whole fish (with a ventral incision) were preserved in RNAlater™ (Ambion Inc., Austin, TX, USA), kept at 4°C for 24 h followed by long-term storage at −80°C. The whole intestine was recovered by dissection when fish were defrosted without gut sectioning, as the gut was not fully developed yet at the end of the stimulus phase. At the end of the challenge phase, pyloric caeca and middle and distal intestines were dissected using a sterilised (ethanol 70%) scalpel and forceps at each gut region midpoint after fat removal (**Additional file 1**) from two fish from each tank (n = 6) and stored in separate vials of RNAlater™ as described above. Anatomically, the distal intestine was distinct and dilated from the middle intestine.

### 2.2 RNA extraction

Total RNA was extracted by homogenising intestinal tissues (20 mg of whole intestine from stimulus and 100–150 mg of intestinal regions from challenge) in 1 mL of TRI Reagent following the manufacturer’s instructions (Sigma, Castleford, UK). The homogenisation process was modified using 3-mm tungsten carbide beads and a TissueLyser II Disruption System (Qiagen GmbH, Hilden, Germany). Following isolation, the RNA was quantified by NanoDrop spectrometry (Thermo Scientific, Waltham, MA, USA), and the integrity was confirmed by Agilent 2100 Bioanalyzer to generate RNA integrity number (RIN) value (which was generally above 9) and then stored at −80°C. Library preparation and sequencing were carried out by a commercial company (Novogene Co., Singapore; https://www.novogene.com/). Briefly, RNA samples were enriched for Poly A+ RNA, used to generate TruSeq libraries, and then sequenced on the NovaSeq platform (PE150) at a depth of 20M sequences per read (40M per sample) with a Q30 score of above 90%.

### 2.3 RNA-seq and statistical analyses

#### 2.3.1 Identifying differentially expressed genes

Datasets (for each phase) were processed using Nextflow (v22.10.7) workflow (35). Data analysis of RNA-seq raw sequences was executed using nf-core rnaseq v3.8 (36) with default parameters on the University of Aberdeen High Performance Computing cluster as described recently (37). Therein, reads were adapter and quality trimmed at default threshold cut-off (Q20) using TrimGalore! v0.6.5 (38) and then aligned to the Ensembl genome and annotation of *Salmo salar* Ssal_v3.1 release 109 (Ssal_v3.1.109, GenBank assembly accession: GCA_905237065.2) using option-aligner star_rsem (39, 40) with STAR (v2.7.10a) index input. Mapped reads were counted using featureCounts [subread v2.0.1 (41)] for gene-level analysis. Quality control checks were carried out at different steps throughout the pipeline using MultiQC [v1.11 (42)]. Differential expression of genes between V vs. M fish for each gut region (whole, middle, and distal) was estimated using DESeq2 (43) in the SARTools R package [v1.7.3 (44)]. The list (complete.txt file) of normalised (by DESeq2) genes was then filtered at a p-value of < 0.01 and |log_2_ fold-change| > 0.1. Genes passing these thresholds were considered as differentially expressed genes (DEGs) as either upregulated (log_2_ fold-change > 0.1) or downregulated (log_2_ fold-change < −0.1) and used for downstream analysis. While pyloric caeca were collected and subjected to RNA sequencing, the observed log_2_ fold-change in gene expression fell below the specified filtration threshold (p < 0.01 and |log_2_ fold-change| > 0.1), rendering them ineligible for further analysis due to their relatively minor alterations.

#### 2.3.2 Gene set enrichment analyses

*S. salar* DEGs (Ensembl gene ids) were annotated against Human Ensembl (GRCh38.p13, GCA_000001405.28) to generate Human Genome Organisation Gene Nomenclature Committee (HGNC) IDs using DIAMOND [v2.0.9.147 (45)] and BioMart (46) and added to **Supplementary Table S1**. DEGs were inputted as HGNC gene identifiers (also termed official gene symbols) into the DAVID 2021 (released December 2021) web user interface (47) separately as either up- or downregulated genes along with all genes before filtering (47) was used as background for gene set enrichment analysis and functional classification of DEGs. DAVID (48, 49) pathway/term categories [gene ontology (GO; with the focus on GO:BP; Biological Process), Kyoto Encyclopedia of Genes and Genomes (KEGG) Orthology (KO), and reactome] were filtered by fold enrichment at a p-value cut-off of < 0.01 (option named EASE on DAVID) and minimum gene count of 3. Other DAVID GO terms (GO:CC; Cellular Process and GO:MF; Metabolic Function) were not the focus of the present study analysis, so in this study, GO was used to refer to GO:BP. Additionally, the list of HGNC IDs and their log_2_ of fold-change were analysed for both (canonical) pathways and upstream drivers using Ingenuity Pathway Analysis (IPA) (50). IPA pathways were filtered to show pathways at −log (p-value) threshold of greater than 1.3 (default in IPA) and z-score greater than 2 (activated pathway) or less than −2 (inhibited pathway). Upstream drivers were displayed at p < 0.01 and |z-score| > 2. Some of the DEGs (**Supplementary Table S1**) that have no HGNC ID equivalent were searched on the Ensembl genome browser [using Ssal_v3.1.110 (51)] and found to be novel genes with uncharacterised functions (genes are hyperlinked as in **Supplementary Table S1**). For further analysis, R v4.3.0 (52) was used. Permutational multivariate analysis of variance (perMANOVA; using adonis2 function) and non-metric multi-dimensional scaling (nMDS; using metaMDS function) from vegan v2.6–4 (53) were performed on Bray–Curtis distances of either unfiltered or filtered normalised counts of DESeq2 to analyse and visualise transcriptomic patterns in the gut of V and M fish in response to first feeding diet. The total number (set size) and unique and shared (intersection size) DEGs in the V vs. M fish comparisons were visualised using UpSet plots [UpSetR v1.4.0 (54)]. GO (specifically GO:BP) analysis in PANTHER [v18.0 (55)] was carried out using the same DAVID HGNC inputs (up- or downregulated gene list along with the background genes) at a false discovery rate (FDR) < 0.05 with aim of a proper interpretation of relevant terms together with the aid of the PANTHER tree-based hierarchical organisation and classification of GO:BP terms. Ensembl gene (bio)types of Atlantic salmon were identified using BioMart [(46), database: Ensembl Genes 110, dataset: Ssal_v3.1, attributes: gene type] on DEGs and unfiltered DESeq2 output genes. Transcript-level analysis was carried out using kallisto [v0.48.0 (56)], and IsoformSwitchAnalyzeR [v2.0.1, default FDR < 0.05 (57, 58)] on the Ssal_v3.1.110 transcriptome (cDNA) and annotation to identify alternative splicing and to predict isoform switches in the V vs. M comparison.

## 3 Results

### 3.1 Fish performance

At the end of the stimulus stage, V fish showed a lower specific growth rate (5.9%/day) than M fish (6.3%/day), whereas at the intermediate stage, M and V fish showed comparable specific growth rates (4.0%/day), feed efficiencies (M fish 1.4 and V fish 1.3), and survival rates (M fish 97.9% and V fish 97.3%). Similarly, after the V challenge phase, comparable specific growth rates (1.8%/day), feed efficiencies (1.0), and survival rates (M fish 100% and V fish 99.6%) were revealed in M and V fish [McMillan et al. (34)].

### 3.2 Transcriptome changes in the intestinal regions

For gene expression analysis, all responses of the gut transcriptome in the V fish (fed the V diet at first feeding) were expressed relative to the M fish (fed the M diet at first feeding), with upregulation and downregulation referring to higher and lower levels of gene expression in V fish than in M fish, respectively.

**Figure 2:**
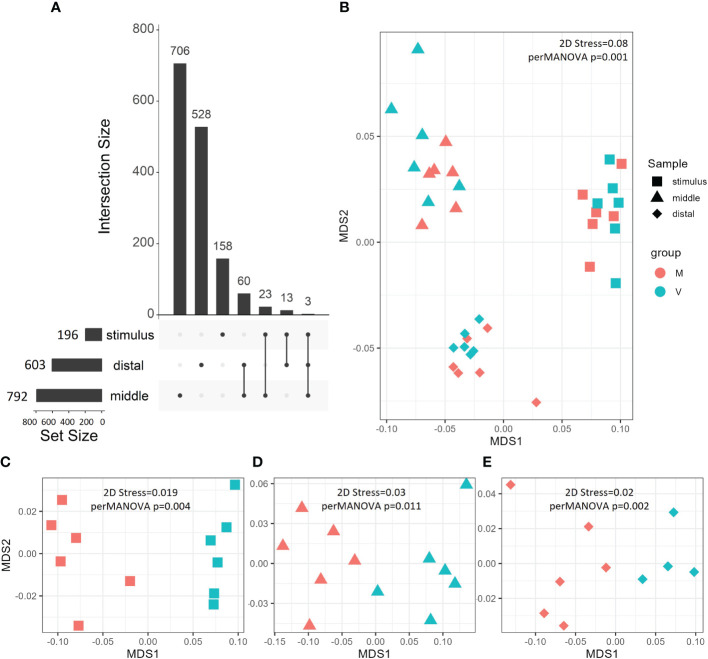
Gene expression patterns visualised by UpSet plot **(A)** and nMDS **(B–E)**. UpSet plots show the total (Set Size) and unique/shared (Intersection Size) DEGs (|log2 fold-change| > 0.1, p < 0.01) between V and M fish gut regions at different phases. nMDS was carried out on Bray–Curtis distances and statistically analysed by perMANOVA on all genes output from DESeq2 **(B)** or filtered DEGs (p < 0.01, |log2 fold-change| > 0.1 **(C–E)**. Gut regions are grouped by shape, while fish are grouped by colour. DEGs, differentially expressed genes; nMDS, non-metric multi-dimensional scaling; perMANOVA, permutational multivariate analysis of variance.

At the end of the stimulus phase, we found 196 significant DEGs (p < 0.01 and |log_2_ fold-change| > 0.1) between V and M fish (**Figure 2A**). At the end of the challenge phase, we found significant 792 DEGs in the middle intestine and 603 DEGs in the distal intestine of V fish compared to M fish (p < 0.01 and |log_2_ fold-change| > 0.1, **Figure 2A**). We found the highest number (**Figure 2A**) and magnitude/fold-change of DEGs (**Table 1**, **Supplementary Table S1**) in the middle intestine when compared to the distal intestine at the end of the challenge phase or the whole intestine at the end of the stimulus phase.

**Table 1:** The top (see **Supplementary Table S1** for all genes) 20 up- or downregulated DEGs (mainly protein-coding) detected against the Atlantic salmon genome after DESeq2 analysis of comparison between V and M fish genes in whole intestine at stimulus and middle and distal intestines at challenge phase.

HGNC ID	Gene Description	ENSEMBL Gene ID	Fold Change	p-value
End of stimulus (whole intestine)				
*RPL9*	ribosomal protein L9	ENSSSAG00000102110	2.39	1.2 ×10^6^
*ELF3*	E74 like ETS transcription factor 3	ENSSSAG00000081244	1.64	2.7×10^7^
*INSIG1*	insulin induced gene 1	ENSSSAG00000068477	1.61	8.2×10^6^
*SLC25A1*	solute carrier family 25 member 1	ENSSSAG00000041957	1.57	2.8×10^5^
*PFKFB4*	6phosphofructo2kinase/fructose2,6biphosphatase 4	ENSSSAG00000041790	1.55	1.5×10^5^
*TKT*	transketolase	ENSSSAG00000095322	1.49	3.7×10^5^
*CPXM1*	carboxypeptidase X, M14 family member 1	ENSSSAG00000072026	1.43	3.4×10^5^
*TNFSF14*	TNF superfamily member 14	ENSSSAG00000082171	1.37	5.6×10^5^
*DHRS3*	dehydrogenase/reductase 3	ENSSSAG00000069479	0.71	4.1×10^5^
*EBF3*	EBF transcription factor 3	ENSSSAG00000045462	0.69	4.0×10^5^
*SREBF1*	sterol regulatory element binding transcription factor 1	ENSSSAG00000003473	0.69	1.2×10^9^
*CCL19*	CC motif chemokine 19	ENSSSAG00000002773	0.58	7.9×10^7^
*BLNK*	B cell linker	ENSSSAG00000080259	0.56	2.6×10^7^
*AGR2*	anterior gradient 2, protein disulphide isomerase family member	ENSSSAG00000073304	0.56	1.43×10^6^
*TRIM22*	tripartite motif containing 22	ENSSSAG00000098249	0.55	8.8×10^6^
*PANK1*	pantothenate kinase 1	ENSSSAG00000113557	0.53	2.3×10^6^
*CALB1*	calbindin 1	ENSSSAG00000058218	0.51	6.7×10^8^
*ALX3*	ALX homeobox 3	ENSSSAG00000074375	0.49	6.0×10^11^
*TEC*	tec protein tyrosine kinase	ENSSSAG00000042383	0.45	1.2 ×10^6^
*HMCN2*	hemicentin 2	ENSSSAG00000114015	0.4	6.0×10^7^
End of challenge (middle intestine)				
*NLRC3*	NLR family CARD domain containing 3	ENSSSAG00000106116	2.42	7.8×10^6^
*IGKV41*	immunoglobulin kappa variable 41	ENSSSAG00000117998	2.39	2.7×10^5^
*IGHV372*	immunoglobulin heavy variable 372*	ENSSSAG00000112422	1.28	1.4×10^4^
*SIRPD*	signal regulatory protein delta	ENSSSAG00000054667	1.24	4.0×10^4^
*SGCE*	sarcoglycan epsilon	ENSSSAG00000047882	1.24	5.6×10^5^
*CYP7A1*	cytochrome P450 family 7 subfamily A member 1	ENSSSAG00000051425	1.22	4.4×10^4^
*IGHV333*	immunoglobulin heavy variable 333*	ENSSSAG00000004153	1.22	3.6×10^4^
*ATP5ME*	ATP synthase membrane subunit e	ENSSSAG00000006812	1.22	3.4×10^6^
*DAPL1*	death associated protein like 1	ENSSSAG00000076051	0.82	2.6×10^7^
*IGKV1D8*	immunoglobulin kappa variable 1D8	ENSSSAG00000048875	0.82	1.8×10^4^
*GGA2*	golgi associated, gamma adaptin ear containing, ARF binding protein 2	ENSSSAG00000004301	0.81	2.0×10^6^
*FOXD3*	forkhead box D3	ENSSSAG00000102893	0.8	3.3×10^4^
*PLPP3*	phospholipid phosphatase 3	ENSSSAG00000073687	0.79	1.8×10^6^
*NCS1*	neuronal calcium sensor 1	ENSSSAG00000066286	0.78	8.1×10^6^
*NMT3*	Nmethyltransferase 3	ENSSSAG00000121379	0.78	6.7×10^5^
*AANAT*	aralkylamine Nacetyltransferase	ENSSSAG00000085086	0.76	5.7×10^5^
*CDH1*	cadherin 1	ENSSSAG00000092585	0.69	1.0×10^5^
*ZP3*	zona pellucida glycoprotein 3	ENSSSAG00000080695	0.68	4.2×10^5^
*HADHB*	hydroxyacylCoA dehydrogenase trifunctional multienzyme complex subunit beta	ENSSSAG00000111749	0.41	2.4×10^7^
*GHRL*	ghrelin and obestatin prepropeptide	ENSSSAG00000045825	0.3	9.9×10^9^
End of challenge (distal intestine)				
*ITGAL*	integrin subunit alpha L	ENSSSAG00000046602	1.73	7.6×10^8^
*URGCP*	upregulator of cell proliferation	ENSSSAG00000003672	1.64	2.7×10^6^
*PLIN3*	perilipin 3	ENSSSAG00000073462	1.52	4.8×10^6^
*NELFCD*	negative elongation factor complex member C/D	ENSSSAG00000109044	1.45	9.6×10^7^
*GLB1*	galactosidase beta 1	ENSSSAG00000051577	1.37	1.0×10^5^
*GLB1*	galactosidase beta 1	ENSSSAG00000058095	1.35	6.6×10^6^
*GPR155*	G proteincoupled receptor 155	ENSSSAG00000121343	1.35	3.1×10^5^
*SDS*	serine dehydratase	ENSSSAG00000074667	1.35	3.0×10^5^
*CBFB*	corebinding factor subunit beta	ENSSSAG00000068137	0.74	6.1×10^6^
*C15orf48*	chromosome 15 open reading frame 48	ENSSSAG00000030104	0.73	2.2×10^5^
*VDR*	vitamin D receptor	ENSSSAG00000007676	0.73	1.7×10^5^
*STX12*	syntaxin 12	ENSSSAG00000056707	0.72	1.5×10^5^
*CTSV*	cathepsin V	ENSSSAG00000008530	0.72	1.0×10^6^
*TACR3*	tachykinin receptor 3	ENSSSAG00000095583	0.71	1.6×10^5^
*SOX12*	SRYbox transcription factor 12	ENSSSAG00000107607	0.7	1.2×10^5^
*RUNX3*	RUNX family transcription factor 3	ENSSSAG00000003444	0.68	2.8×10^7^
*EPAS1*	endothelial PAS domain protein 1	ENSSSAG00000030127	0.64	5.1×10^6^
*SLC1A7*	solute carrier family 1 member 7	ENSSSAG00000015840	0.63	1.2×10^6^
*COX6A1*	cytochrome c oxidase subunit 6A1	ENSSSAG00000041369	0.58	2.6×10^7^
*FABP2*	fatty acid binding protein 2	ENSSSAG00000002671	0.55	1.7×10^7^

Genes were selected based on p-value < 0.01 and those showing the greatest up- or downregulation in relation to fold-change.

DEGs, differentially expressed genes; HGNC, HUGO (Human Genome Organisation) Gene Nomenclature Committee.

*Ig V gene.

In between gut regions, we then analysed common and unique DEGs between the gut (whole at the end stimulus phase and the middle and distal intestines at the end of the challenge phase) (**Figure 2A**). The common DEGs between the middle and distal intestines compose the largest intersection (60 DEGs), indicating the presence of a common gut dietary signature at least at the end of the challenge. While whole intestine at the end of the stimulus phase uniquely shared 23 and 13 DEGs with the middle and distal intestines, respectively, at the challenge phase, indicating a lesser number of shared DEGs across developmental stages than between gut regions (**Figure 2A**).

To analyse transcriptomic patterns of different gut regions at the stimulus and challenge phases, perMANOVA of the unfiltered normalised DESeq2 counts showed significant differences between the whole, middle, and distal intestines (p = 0.001). Additionally, perMANOVA of DEGs showed significant differences between V and M fish in the whole intestine at the end of stimulus (p = 0.004) and the middle (p = 0.011) and distal (p = 0.002) intestines at the end of the challenge. These group differences were visualised using nMDS between gut regions (stress = 0.08, **Figure 2B**) and between V and M fish in stimulus whole intestine (stress = 0.019, **Figure 2C**) and challenge middle (stress = 0.02, **Figure 2D**) and distal (stress = 0.03, **Figure 2E**) intestines.

The focus of further analysis was, therefore, to characterise gut transcriptomic responses to the V diet, after either stimulus (short-term effects of diet) or challenge (long-term effects related to potential programming), and determine whether the observed effects at stimulus persisted or other effects were present in the V fish after challenge. As the M diet had a standard composition, M fish were used as a reference to which we compared the V fish. All V vs. M fish comparisons were carried out at gene and gene set enrichment analysis levels, while the transcript-level analysis showed no significant transcript/isoform switches, although alternative splicing events were detected (data not shown).

#### 3.2.1 Transcriptomic responses in V vs. M fish at stimulus

**Figure 3:**
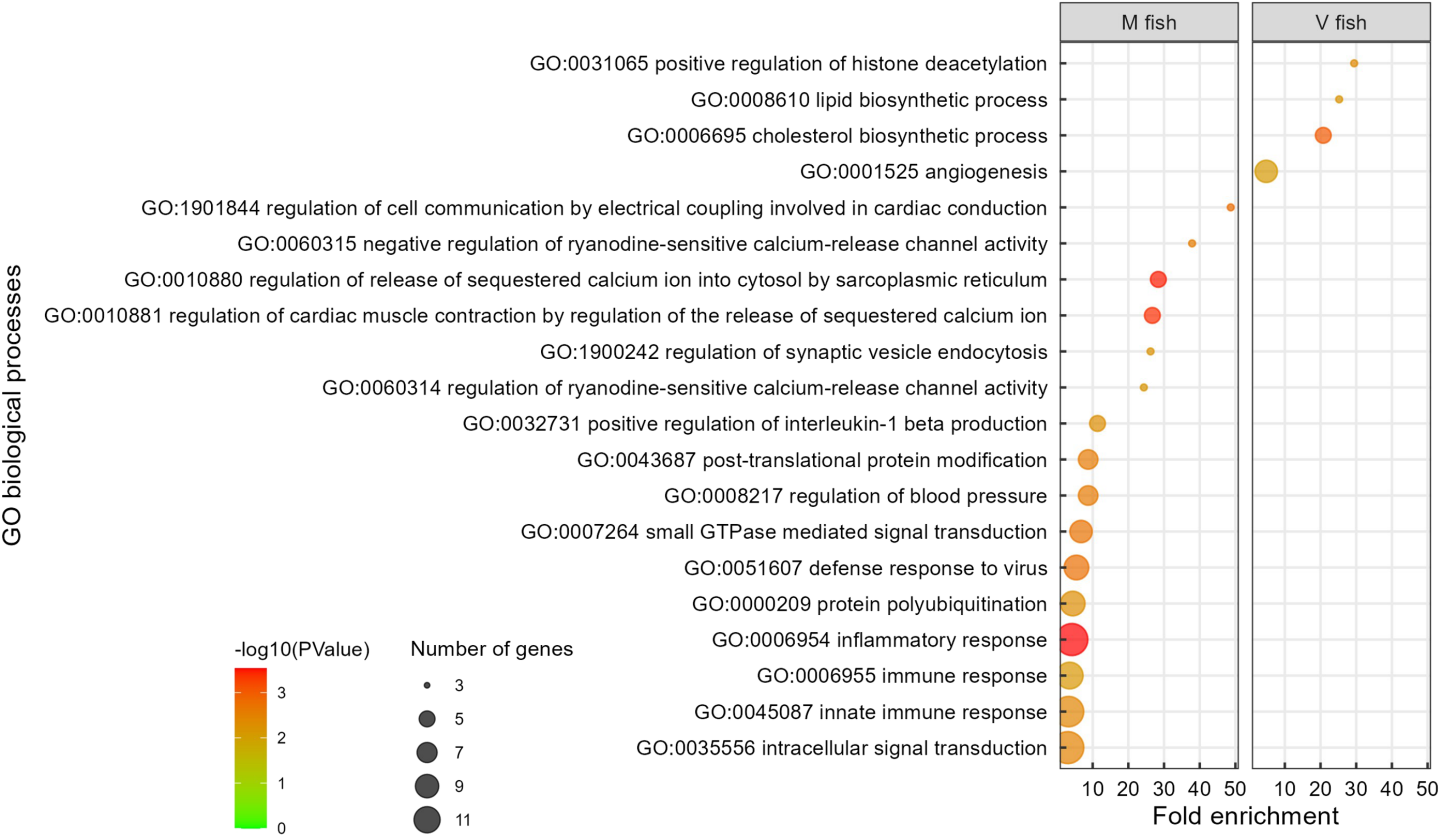
Gene ontology analysis of V fish vs. M fish in whole intestine at stimulus phase. GO terms (at level of biological processes) have been filtered to show DEGs involved within each pathway greater than 3 counts (represented by the size of the dot) and a p-value of less than 0.01. Colours represent −log10 (p-value), with red being the highest. GO, gene ontology; DEGs, differentially expressed genes.

Among the four significantly enriched GO terms altered significantly in V fish compared to M fish at the end of stimulus, “histone deacetylation” was the most enriched GO term (p < 0.01, **Figure 3**, **Supplementary Table S2A**). Lipid biosynthetic process and cholesterol biosynthetic process were the next highly enriched GO terms (p < 0.01, **Figure 3**, **Supplementary Table S2A**). Consistent with this GO term enrichment, there were also changes in reactome pathways (p < 0.01, **Supplementary Figure S1**, **Supplementary Table S2A**) mapped to “metabolism of lipids” and “fatty acyl-CoA biosynthesis pathway”, an intermediate in lipid metabolism, in addition to the “regulation of cholesterol biosynthesis by *SREBP*” pathway (p < 0.01, **Supplementary Figure S1**, **Supplementary Table S2A**). Sterol regulatory element binding proteins (*SREBP*) are a family of transcription factors that regulate enzymes involved in lipid homeostasis including endogenous cholesterol, fatty acid, triacylglycerol, and phospholipid synthesis. The final enriched GO term in V fish was “angiogenesis”, but whether it is positively or negatively regulated was not clarified (p < 0.01, **Figure 3**, **Supplementary Table S2A**).

**Figure 4:**
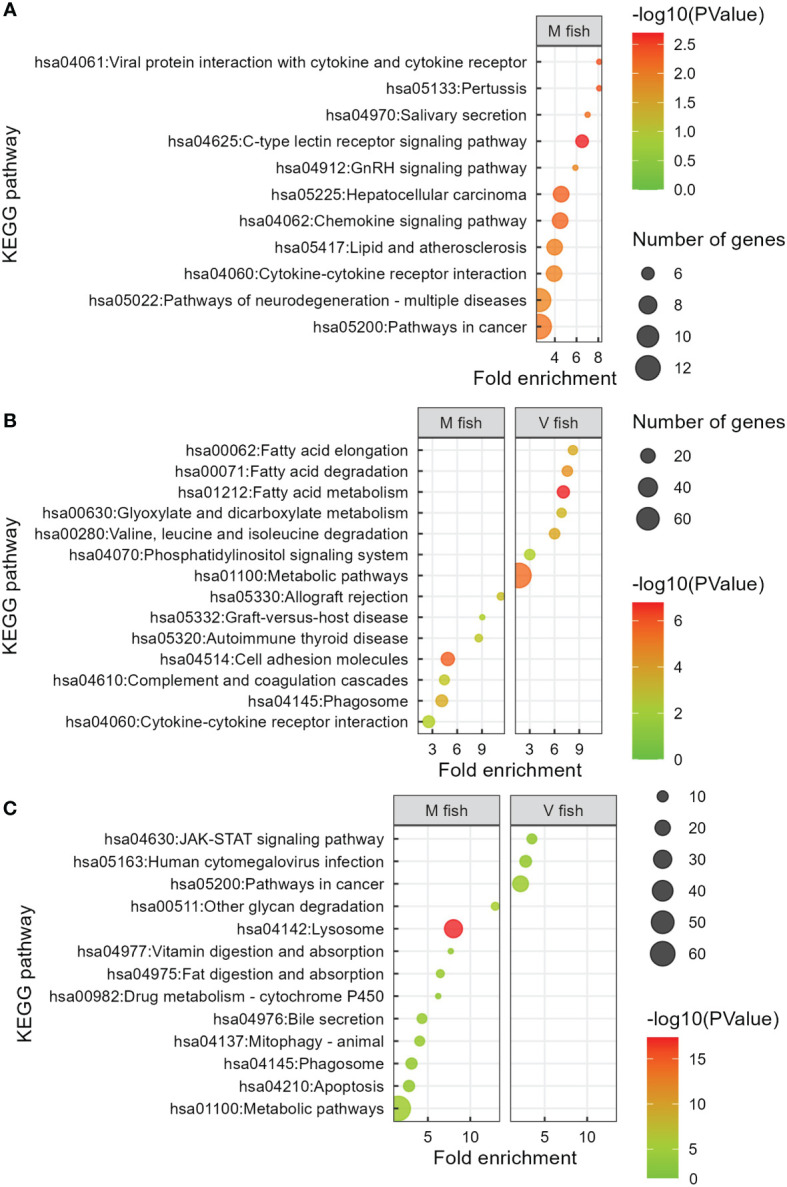
KEGG analysis of V fish vs. M fish in whole intestine at stimulus phase **(A)** and middle **(B)** and distal **(C)** intestines at challenge phase. KEGG pathways have been filtered to show DEGs involved within each pathway greater than 3 counts (represented by the size of the dot) and a p-value of less than 0.01. Colours represent −log10 (p-value), with red being the highest. KEGG, Kyoto Encyclopedia of Genes and Genomes; DEGs, differentially expressed genes.

In contrast, 16 significant GO terms were enriched in M fish including homeostasis, signalling, immune response (mainly innate, including *IL-1β* positive regulation), and inflammatory response to the virus (p < 0.01, **Figure 3**, **Supplementary Table S2B**). Moreover, post-translational protein modification and protein polyubiquitination terms were enriched in M fish and are known to be involved in post-translational processes. The enriched GO terms in M fish were generally consistent with analyses of KEGG pathways (p < 0.01, **Figure 4**, **Supplementary Table S2B**), reactome (p < 0.01, **Supplementary Figure S1A**, **Supplementary Table S2B**), and IPA (−log(p) > 1.3, **Figure S2**). “NRF2-mediated oxidative stress response” is an IPA-enriched pathway in M fish when compared to V fish, which promotes cell survival by reducing oxidative stress by reactive oxygen species (ROS) (−log(p) > 1.3, **Supplementary Figure S2**). Additionally, the IPA pathway “Cholecystokinin/Gastrin-mediated signalling” was enriched in M fish (−log(p) > 1.3, **Supplementary Figure S2**).

“Upstream regulator” refers to any transcriptional molecule that can explain downstream gene expression (59). The upstream regulators, as predicted by IPA analysis, that were activated and proposed to drive the significant changes in V fish gut included regulators of lipid metabolism such as *PPARG*, *MLXIPL*, and *SREBF2* (p < 0.01, **Supplementary Table S3A**). *PPARG* is a nuclear receptor that plays a role in the regulation of adipocyte differentiation and lipid metabolism. *MLXIPL* and *SREBF2* are both transcription factors, with *MLXIPL* involved in the regulation of glucose metabolism and lipogenesis, whereas *SREBF2* regulates cholesterol homeostasis. The significantly inhibited regulators in V fish were mainly signalling and immune responses, including *IL-1*, lipopolysaccharide (LPS), and *IRF7* (p < 0.01, **Supplementary Table S3B**). LPS is a component of the cell wall of gram-negative bacteria that can trigger (innate) immune responses by binding to the receptor *TLR4*. *IRF7* is a transcription factor involved in the regulation of interferon signalling. Those predicted activated and inhibited upstream regulators are in line with the enriched GO terms mentioned above.

#### 3.2.2 Transcriptomic responses in V vs. M fish in middle intestine at challenge

**Figure 5:**
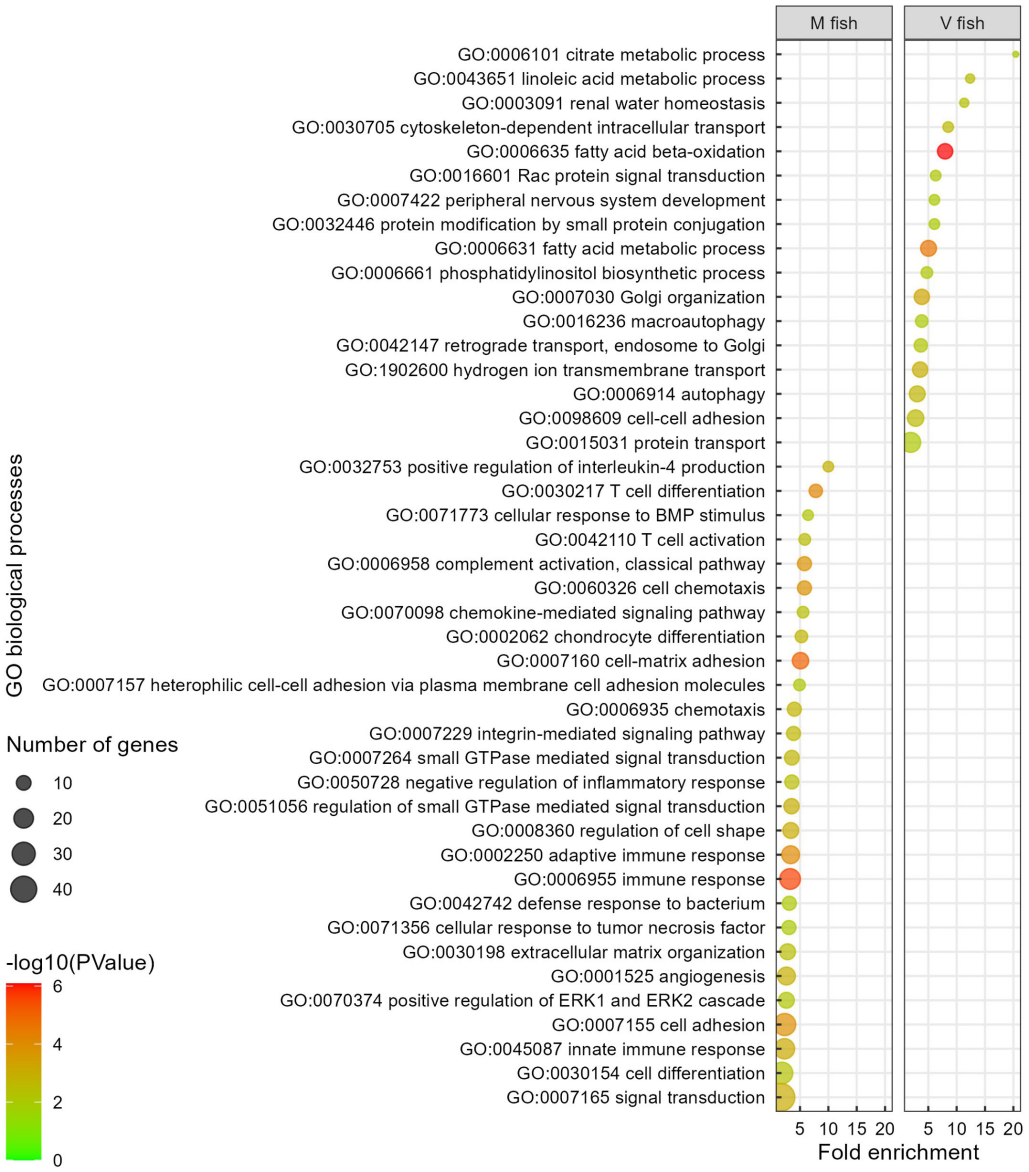
Gene ontology analysis of V fish vs. M fish in middle intestine at challenge phase. GO terms (at level of biological processes) have been filtered to show DEGs involved within each pathway greater than 3 counts (represented by the size of the dot) and a p-value of less than 0.01. Colours represent −log10 (p-value), with red being the highest. GO, gene ontology; DEGs, differentially expressed genes.

Overall, with regard to gene set enrichment [using DAVID (GO, KO, and reactome), and IPA], enriched functions could be broadly grouped into dietary metabolism and energy generation, membrane dynamics, detoxification, and tissue remodelling in the middle intestine of V fish. Fatty acid metabolism functions included “linoleic acid metabolic process”, “fatty acid beta-oxidation”, and “fatty acid metabolic process” GO terms (p < 0.01, **Figure 5**) (KEGG: “fatty acid elongation”, “fatty acid degradation”, and “fatty acid metabolism”, p < 0.01, **Figure 4B**) (IPA: “superpathway of cholesterol biosynthesis”, −log(p) > 1.3, **Supplementary Figure S3A**). Out of the 20 enriched reactome pathways, 11 pathways (p < 0.01, **Supplementary Figure S1B**) were categorised as “fatty acid metabolism” or “metabolism of lipids” that occur in endoplasmic reticulum (including “fatty acyl-CoA biosynthesis”, “synthesis of very long-chain fatty acyl-CoAs”) or mitochondria (six mitochondrial fatty acid beta-oxidation pathways). Furthermore, GO terms “cytoskeleton-dependent intracellular transport”, “hydrogen ion transmembrane transport”, “cell–cell adhesion”, “phosphatidylinositol biosynthetic process” (p < 0.01, **Figure 5**), and KEGG “phosphatidylinositol signalling system” (p < 0.01, **Figure 4B**) could be categorised as involved in cell signalling, membrane dynamics, and vesicle trafficking. Similarly, IPA pathways included “RHOA signalling”, “superpathway of inositol phosphate compounds”, and “D-myo-inositol(1,4,5)-trisphosphate biosynthesis” (−log(p) > 1.3, **Supplementary Figure S3A**). The reactome pathways (p < 0.01, **Supplementary Figure S1B**) included “beta-catenin independent WNT signalling”, “MAPK family signalling cascades”, “phospholipid metabolism”, “Ca^2+^ pathway”, and “transport of small molecules”. For protein metabolism and energy production, “valine, leucine and isoleucine degradation” pathways associated with Krebs/TCA cycle (“glyoxylate and dicarboxylate metabolism” and “citrate metabolic process”) in addition to “Golgi organisation”, “protein transport”, and “retrograde transport, endosome to Golgi” were enriched GO/KEGG in the middle intestine of V fish (p < 0.01, **Supplementary Table S2C**). Golgi apparatus is an organelle responsible for protein degradation, modifying (for quality control), sorting, packaging proteins for transport, and post-translational protein processing (GO: “protein modification by small protein conjugation” and “Rac protein signal transduction”, p < 0.01, **Figure 5**) (reactome: “RAS processing”, p < 0.01, **Supplementary Figure S1C**). Additionally, enriched pathways were involved in cellular stress and death including “programmed cell death” and “apoptosis” (reactome, p < 0.01, **Supplementary Figure S1C**), plus “macroautophagy” (GO, p < 0.01, **Figure 5**), “xenobiotic metabolism AHR signalling pathway”, and “NRF2-mediated oxidative stress response” (IPA, −log(p) > 1.3, **Supplementary Figure S3B**). Furthermore, one of the highly enriched pathways in V fish was “renal water homeostasis”. Additionally, “developing peripheral nervous system” was one of the highly enriched GO terms in V fish.

The GO terms and pathways (DAVID and IPA) assessed as being decreased in activity in the middle intestine of V fish (**Figure 5**, **Supplementary Table S2D**) were related to immune (innate and adaptive) and inflammatory response and immune signalling, cell–cell communication, cellular adhesion and cell signalling, and tissue degeneration. As GO terms in DAVID (p < 0.01, **Supplementary Table S2**) and PANTHER (FDR < 0.05, **Additional file 2**) showed very similar results, interestingly, enriched PANTHER GO terms and DAVID KEGG pathways indicated high severity of immune and inflammatory responses including “acute inflammatory response” (GO, FDR < 0.05, **Additional file 2**) and “allograft rejection”, “graft-versus-host disease”, and “autoimmune thyroid disease” (KEGG, p < 0.01, **Figure 4**) in M fish. The severity of inflammatory response in M fish was reflected in degeneration and a higher need for tissue repair as represented by pathways: “degradation of the extracellular matrix” (reactome, p < 0.01, **Supplementary Figure S1**) and “extracellular matrix organisation” and “angiogenesis” (GO, p < 0.01, **Figure 5**), and “HIPPO signalling” (the fundamental regulatory network that controls cell growth and organ size, IPA, −log(p) > 1.3, **Supplementary Figure S3A**).

#### 3.2.3 Transcriptomic responses in V vs. M fish in distal intestine at challenge

**Figure 6:**
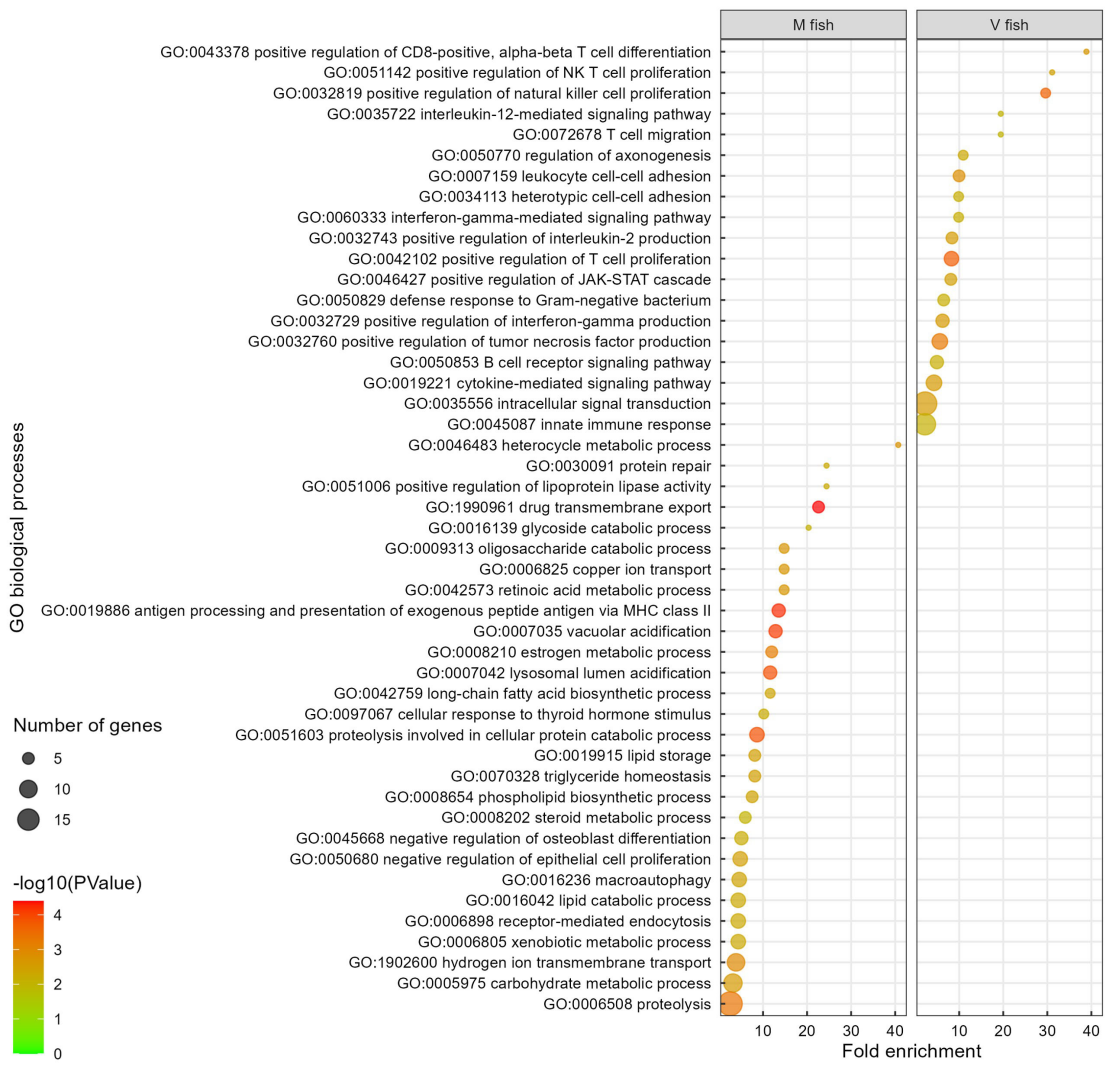
Gene ontology analysis of V fish vs. M fish in distal intestine at challenge phase. GO terms (at level of biological processes) have been filtered to show DEGs involved within each pathway greater than 3 counts (represented by the size of the dot) and a p-value of less than 0.01. Colours represent −log10 (p-value), with red being the highest. GO, gene ontology; DEGs, differentially expressed genes.

Induced changes in the distal intestinal transcriptome of V fish, represented in this study by 17 enriched GO terms, are all related to immune response and signalling pathways (**Figure 6**, **Supplementary Table S2E**). Fewer pathways in KEGG (**Figure 4**, **Supplementary Table S2E**), reactome (**Supplementary Figure S1**, **Supplementary Table S2E**), and IPA (**Supplementary Figure S3**) analyses showed similar enriched pathways in V fish. *IL-12* is a cytokine involved in signalling immune response (*IL-12*-mediated signalling pathway, p < 0.01, **Figure 6**) and can activate the “JAK-STAT signalling pathway” (KEGG, **Figure 4B**), promoting T-cell differentiation (CD4^+^ and CD8^+^), “positive regulation of IFN‐γ production” (GO, p < 0.01, **Figure 6**), and “crosstalk between dendritic cells and NK cells” (IPA, −log(p) > 1.3, **Supplementary Figure S3A**). For GO enrichment using PANTHER (FDR < 0.05, **Additional file 2**), similar GO terms to DAVID were identified with “positive regulation of CD8-positive, alpha-beta T-cell differentiation”, being enriched GO terms in V fish in both DAVID and PANTHER, while GO term “CD4-positive, alpha-beta T-cell differentiation” was enriched only in PANTHER.

In M fish, the 27 enriched GO terms play roles in cellular processes including digestion, lipid and carbohydrate metabolism, cellular transport and absorption/trafficking, signalling, and stress and immune responses (p < 0.01, **Figure 6**, **Supplementary Table S2F**). Several GO terms and pathways were involved in lipid and fatty acid metabolism including GO “long-chain fatty acid biosynthetic process”, “lipid storage”, “triglyceride homeostasis”, “positive regulation of lipoprotein lipase activity”, “heterocycle metabolic process”, and “phospholipid biosynthetic process” terms (p < 0.01, **Figure 6**), and KEGG “bile secretion” and “lipid digestion and absorption” pathways (p < 0.01, **Figure 4B**). GO terms involved in carbohydrate and energy metabolism included “oligosaccharide catabolic process”, “glycoside catabolic process”, “carbohydrate metabolic process”, and “cellular response to thyroid hormone stimulus”. Moreover, cellular stress, tissue degeneration and regulation of proliferation were represented in GO terms “proteolysis involved in cellular protein catabolic process”, “macroautophagy”, “lysosomal lumen acidification”, “vacuolar acidification” and “receptor-mediated endocytosis”, “lipid catabolic process”, “protein repair, and “negative regulation of epithelial cell proliferation” (p < 0.01, **Figure 6**), plus KEGG “lysosome”, “phagosome”, “apoptosis”, “mitophagy”, “other glycan degradation” (p < 0.01, **Figure 4B**), and IPA “MSP-RON signalling in macrophages pathway” (−log(p) > 1.3, **Supplementary Figure S3A**) pathways, respectively. Additionally, detoxification and elimination of foreign compounds via “drug transmembrane export” and “xenobiotic metabolic process” were enriched in M fish (GO, p < 0.01, **Figure 6**) (KEGG: drug metabolism—cytochrome P450, p < 0.01, **Figure 4B**). “Retinoic acid metabolic process” is part of vitamin digestion and absorption that help in signalling (**Figure 6**) (KEGG: “vitamin digestion and absorption”, p < 0.01, **Figure 4B**) (IPA: “VDR/RXR signalling”, −log(p) > 1.3, **Supplementary Figure S3A**), while “copper ion transport” and “hydrogen ion transmembrane transport” are involved mainly in transport (p < 0.01, **Figure 6**). Furthermore, “antigen processing and presentation of exogenous peptide antigen via MHC class II” (p < 0.01, **Figure 6**) (reactome: “MHC class II antigen presentation”, p < 0.01, **Supplementary Figure S1B**) was enriched in M fish.

The upstream activated regulators predicted by IPA in V fish were consistent with the enriched GO terms and pathways in V fish including *IL-12* and *CD28*, co-stimulatory molecules of T cells that can regulate genes involved in T-cell activation pathways (p < 0.01, **Supplementary Table S3E**). However, the IPA drivers that were found to be significantly activated in M fish (i.e., inhibited in V fish, p < 0.01) included genistein, a naturally occurring isoflavone found in soy products, known to have estrogenic properties, and β-estradiol, an oestrogen hormone. NFE2L2, a transcription factor that regulates gene expression involved in cellular protection against oxidative stress and detoxification of harmful compounds, was one of the drivers and in line with the stress response-based GO terms and pathways upregulated in the distal intestine of M fish (see **Supplementary Table S3F** for drivers).

## 4 Discussion

This study hypothesised that feeding Atlantic salmon with a plant-rich diet at first feeding will have long-term effects on their nutritional physiology. The rationale for this study is that the fish digestive system is not fully developed during first feeding (60), a critical developmental stage that is highly plastic when responding to stimuli such as diet (61, 62). It is also a crucial stage where the commensal microbiome is established and the immune system in the fish matures (63–65). To investigate the impact of NP, we followed a classical NP design (16) where Atlantic salmon were fed a plant-rich diet for 2 weeks as a nutritional stimulus (V fish) versus a control/standard, marine-based diet (M fish). Then, both M and V fish were fed the M diet as an intermediate period before all fish were challenged with a plant-rich diet similar to that which V fish had been exposed to at first feeding. The aim of the first feeding stimulus with a plant-rich diet for a short term is to elicit NP. This period was chosen as short enough not to expose Atlantic salmon to a V diet for a longer period during this critical stage that may cause negative effects. For example, Clarkson et al. (20) found increased inflammatory responses in triploid Atlantic salmon after the challenge that was originally stimulated for 3 weeks with soy protein concentrates. Still, a long enough period is required to allow fish to adapt to this type of feed ingredients during this plastic developmental stage, particularly with respect to digestive tract development. The nutritional window timing and length along with dietary composition are crucial for NP to provide a positive physiological response, which needs further research (16). We designed a 6-week challenge phase, as this period was enough to see phenotypic differences with a 3-week NP window at first feeding using similar diets (20) as in our study, in addition to a follow-up NP study on the samples from Clarkson et al. (20) study with the same experimental design and demonstrated molecular (transcriptomic and epigenetic) hepatic responses (22).

To investigate how different gut regions responded after NP, which has not been studied yet, we segmented the gut into regions (pyloric caeca and middle and distal intestines) after the challenge, but the development stage of the gut at the end of stimulus (2 weeks post-first feeding) hindered us from obtaining gut regions. We did not consider pyloric caeca, as they did not pass the current study DEG filtration threshold (p < 0.01 and |log_2_ fold-change| > 0.1); thus, they were removed from further analyses (no enriched pathways were retrieved, eventually). Transcriptomic responses of the whole intestine at the end of the stimulus phase and both the middle and distal intestines at the end of the challenge phase were analysed for gene set enrichment to obtain pathways that predict potentially changed physiological function. Although there was no improved growth performance after the V challenge, we detected differential transcriptome changes after NP in gut regions. Several biological processes were significantly enriched in the V fish following stimulus relating to intestinal processes including enriched lipid metabolism and positive epigenetic regulation, and downregulated immune-related processes. The middle intestine of V fish and M fish at challenge showed enriched lipid metabolism and acute immune response pathways, respectively. In contrast to the intestine at stimulus, the distal intestine of V fish at the challenge phase exhibited enriched immunomodulatory response pathways, while the distal intestine of M fish indicated pathways related to tissue degeneration and detoxification along with lipid metabolic processes.

### 4.1 Gene set enrichment suggested that both epigenetic and immunoregulatory processes were altered following diet manipulation

#### 4.1.1 Activated histone deacetylation and inhibited immune responses in V fish at stimulus

The whole intestine at the end of the stimulus phase revealed a systemic response of positive regulation of histone deacetylation, suggesting that epigenetic regulatory changes may have happened mainly at the early plastic developmental stage where the *HDAC8* gene (histone deacetylase 8) was upregulated. The profile of immune responses that were downregulated in V fish included positive regulation of *IL-1β* production, inflammatory response, and innate immune response. These responses were linked with upregulated *HCAR2* gene (hydroxycarboxylic receptor 2) and downregulated genes: interleukin-10 (*IL-10*) receptor and *GBP3* (known in *S. salar* as interferon-induced guanylate-binding protein 1-like). These results of immune attenuation could be the result of the associated post-translational processing of (immune-related) proteins in response to the V diet. This immune profile (**Figure 3**) may also be attributed to a metabolic product of the microbiome, butyrate, reported to be associated with increased *IL-10* in Atlantic salmon and *HCAR2* in RTgutGC cells, and decreased *IL-1β*, *IFN-α*, and viral load in *SHK-1* cells (66). Butyrate is a short-chain fatty acid found in the gut originating from feed (67) and/or synthesised by microbiota (66) from complex carbohydrates found mainly in plant-based diets (68). Furthermore, although butyrate and other short-chain fatty acids are reported to inhibit *HDAC*s (66), *HDAC8* was upregulated in the present study in V fish, which may suggest that other factors were strengthening the *HDAC8* expression signal supporting early-life high plasticity. Additionally, after challenge, *CECR2* (histone acetyl-lysine reader), *HDAC7* (histone deacetylase 7), and *BCL11B* (BAF chromatin remodelling complex subunit *BCL11B*) were upregulated in the distal intestine of V fish with no downregulated histone-related genes. In contrast, upregulated *SAP30L* (known in *S. salar* as histone deacetylase complex subunit *SAP30L*) and downregulated *EZH1* (enhancer of zeste 1 polycomb repressive complex 2 subunits) were identified in the middle intestine. Taken together, these findings could be associated with potential epigenetic modifications (as markers) that regulate metabolic and immune alterations during the challenge phase. Interestingly, the gene that showed the greatest upregulation in the middle intestine of V fish has nucleic acid binding functionality (**Supplementary Table S1C**), supporting the possibility of epigenetic modification of metabolic gene expression in the middle intestine. A previous study in medaka (*Oryzias latipes*) found a high-fat diet in early life produced extensive changes in the transcriptome, chromatin accessibility, and histone modifications of metabolic genes in the liver that were reversed at the adult stage, while other changes related to “cell signalling” genes were non-reversible (69). In the current study, no transcript/isoform switches were detected for the same gene between V and M fish after either feeding phase, implying potentially stable gene function and regulation in V fish when compared to M fish. However, whether the epigenetic changes persisted or reversed at the challenge phase compared to the stimulus phase requires further investigation. This is especially relevant given that dietary modifications can initially induce subtle epigenetic alterations but later have significant effects on subsequent immune function, as only a twofold increase in methylation can lead to physiological changes (70). To summarise, epigenome-level adaptations are considered a putative mechanism for capturing early-life nutritional genomic imprinting (69).

#### 4.1.2 Activated potential immunotolerance in middle and distal intestines of V fish at challenge phase

At the end of the challenge phase, immune responses induced in the distal intestine of V fish were particularly regulatory. Enriched pathways associated with CD4^+^ and CD8^+^ T-cell differentiation, NK cell activation and proliferation, T-cell activation, proliferation and migration, and B-cell differentiation were identified. Positive regulation of *IL-2*, *IFNγ*, and *TNF* proinflammatory cytokine production was found, and the JAK-STAT signalling cascade was also enriched along with signalling mediated by cytokines (*IL-2*, *IL-12*, and *IFNγ*) and B-cell receptor. *IFN-γ* is produced not only by NK cells (innate) but also by CD4^+^ Th and CD8^+^ Tc adaptive immune cells (71). However, in rainbow trout, CD4 and CD8 molecules are linked not only to T cells but also to other cells, including dendritic cells on intestinal mucosa (72) and monocytes/macrophages (73). Although not as well studied as their mammalian counterparts, CD4^+^ and CD8^+^ T cells and NK cells are believed to play an active role in mucosal immunity in fish (74). Innate immunity is known to instruct adaptive immunity of invading insults and how to deal with them, which results in a feedback loop regulating immune response (74, 75). Although T and B cells are associated mainly with the adaptive immune response, it is suggested that B cells also contribute to innate immunity in mucosal surfaces (71). *IL-2* is a central cytokine in coordinating the development and survival of T-regulatory (Treg) cells, thus regulating immune tolerance (76), while its absence caused an autoimmune response (77). However, *IL-12* is one of the repressors of immune tolerance orchestrating with *IL-2*, the main immunotolerance activator in tolerance homeostasis as previously reported (76, 78). Although *FOXP3*, a T cell-specific transcription factor, is key in (CD4^+^) Treg cell development in Atlantic salmon (79), *FOXP1*, an upregulated DEG in the distal intestine of V fish, has been found in mammals and mice to play important roles in regulating Treg immunosuppressive function and coordinating quiescent Treg cells (80–83). Upregulation of *FOXP1* in the present study supports T cells in the distal intestine being mainly inducible Treg that are critically important in maintaining tolerance and preventing autoimmunity. Immunotolerance, the ability to prevent unwanted inflammation, maintain immune homeostasis, and unwanted reactions to endogenous host molecules or harmless antigens (84), is also supported by the highest enriched KEGG autoimmune pathways in the middle intestine of M fish, but not in V fish (distal or middle intestine). KEGG pathway enrichment also displayed apoptosis, programmed cell death, and macroautophagy in both the middle and distal intestines of M fish, but not V fish (**Figures 4B,C**), consistent with the notion that V fish maintained immunotolerance and homeostasis during digestion and absorption. Furthermore, maintaining immune tolerance and enforcing barrier functions are some of the diverse functional contributions of a positively regulated JAK-STAT cascade (85) found in the distal intestine of V fish. In addition, MAPK signalling, which was enriched in the middle intestine of V fish, could help in activating downstream pathways involved in physiological acclimation and tolerance against stressors (86). Finally, the enriched “developing peripheral nervous system” in the middle intestine of V fish may be part of neuroimmune regulation via the brain–gut axis, previously reported in rainbow trout and Atlantic salmon (87, 88).

### 4.2 Enriched immune pathways at challenge after first exposure of M fish to plant-rich diet

At the end of the challenge phase, M fish that had not been previously exposed to the V diet were found to have a number of enriched immunological processes in the middle intestine, including autoimmune (**Figure 4B**), adaptive immune (**Figure 5**), and also distal intestine acute immune responses (**Additional file 2**) along with oxidative stress, tissue degeneration, and detoxification. The highest enriched pathway, positive regulation of *IL-4* production, along with *TGFBI* (transforming growth factor beta induced), suggest that the V stimulus promoted immune-suppressive activity, which in mammals is associated with Th9 cells linked to allergy in asthma and autoimmunity (71, 89). Th9 (derived from reprogrammed Th2) can also release *IL-4*, activating the *IL-4* ^+^ Tfh (a CD4^+^ T-cell subset) that, via the *IL-4* mediated signalling pathway, leads to B-cell activation and switching antibody isotopes to produce IgE (89–92). Immunoglobulins (protein coding or identified Ig V gene type) are highly upregulated particularly in the middle intestine of M fish (**Table 1**), potentially indicating increased activity of B cells. The current study also found a potential increase in oxidative stress, as *TMIGD1* (known in *S. salar* as transmembrane and immunoglobulin domain containing 1), a toxicity biomarker of redox disturbance, was upregulated in the distal intestine of M fish. Upregulation of *TMIGD1* was found in zebrafish with hepatic damage (93). Furthermore, *CD40* was upregulated in M fish’s middle intestine, suggesting an increase in antigen processing and presentation, helping with B-cell activation and antibody production (94). Also, *CD40* has been reported to activate gut epithelial cells to act as immune effector cells and release pro- and anti-inflammatory mediators (95). Additionally, digestive metabolic processes were upregulated predominantly in the distal intestine of M fish rather than the middle intestine, suggesting possibly extended/delayed digestive metabolism in the gut preserving energy for immune response and increasing time to deal with the new diet (V diet, **Figures 5**, **6**).

### 4.3 Differences in metabolic adaptation of V and M fish to V diet following programming

Compared to M fish, V fish at stimulus showed an increase in fatty acid and cholesterol metabolic processes, suggesting enhanced metabolism of V diet with its different lipid profile (lower omega-3 and higher omega-6 fatty acid levels) from the standard M diet. Generally, marine-based diets have higher omega-3 and lower omega-6 fatty acids than other aquatic alternative diets (plant- or insect-based diets) (96). While at the challenge, in the V fish, the middle intestine had enriched pathways for fatty acid beta oxidation, and lipid degradation and elongation, suggesting improved lipid metabolism compared to M fish. Similarly, a study using vegetable oil as an early nutritional stimulus (for 30 days) in a large yellow croaker (*Larimichthys crocea*) reported increased expression of lipogenesis-related (*acc1*) and LC-PUFA synthesis genes (*△6fad* and *elovl4*) in the liver compared to the fish oil nutritional history after 30-day challenge (97). Improved functionality in V fish is further supported by the enrichment of linoleic acid metabolic process in the middle intestine compared to M fish at challenge, at which stage dietary linoleic acid was the same in both groups of fish. Moreover, dietary history differs in M and V fish since linoleic acid was higher in V than in M diet at stimulus, which may be a contributing factor for programming. Whether the high content of precursors for lipid biosynthesis (omega-6 fatty acids) or the low levels of omega-3 fatty acids are the trigger for the nutritional programming phenomena is not yet fully investigated (98). However, in the current study, the dietary composition of omega-3 fatty acids was not lower than the minimum requirements in freshwater Atlantic salmon as recently studied (99). In the current study, the enriched pathways in the distal intestine of V fish suggest that the regulated immune responses are for immune protection that naturally occurs during trafficking (100, 101). This is in agreement with other studies where nutrient absorption (also termed trafficking) (102, 103) and immune defence processes (104, 105) appear to increasingly occur towards the distal intestine of fish. In contrast to V fish, M fish had additional processes that were altered including “lipid digestion” and “tissue degeneration and detoxification” during trafficking, which could indicate delayed/extended digestive physiology. For lipids, this is supported by evidence that lipolytic digestive action is the highest in the proximal intestine and decreases towards the distal intestine (106), whereas gene expression indicates the opposite is seen in M fish. However, further metabolic investigations are required to confirm the mechanisms of action in nutritional programming.

## 5 Concluding remarks

The present study has demonstrated that early nutritional history during the plastic developmental stage of first feeding in Atlantic salmon can impact the gut whole transcriptome in both short- and long-term. The current work expands on the previously reported metabolic processes affected in response to nutritional programming, particularly in the gut. The data have indicated acute inflammatory responses in fish exposed for the first time to a plant-based diet. Those fish that had been fed a plant-based diet in early life showed reduced immunity and inflammation during first feeding and signs of immunotolerance later in life (after challenge) that potentially improved the functioning of the gut. While the mechanism for this response remains unclear, the results identified enriched pathways and upregulated differentially expressed genes likely associated with epigenetic modification of DNA after early nutritional stimulation by plant-based diets, which may play a role in gene regulation in later life, which requires further research.

## Data availability statement

The datasets presented in this study can be found in online repositories. The names of the repository/repositories and accession number(s) can be found below: https://www.ncbi.nlm.nih.gov/bioproject/PRJNA1062032/ (BioSample RNA accession numbers: SAMN39285423-70).

## Ethics statement

The animal study was approved by UoS Animal Welfare and Ethical Review Board. The study was conducted in accordance with the local legislation and institutional requirements.

## Author contributions

MT: Data curation, Formal Analysis, Funding acquisition, Investigation, Methodology, Software, Validation, Visualization, Writing – original draft, Writing – review & editing. MB: Conceptualization, Funding acquisition, Investigation, Methodology, Resources, Supervision, Writing – review & editing. StM: Investigation, Methodology, Resources, Writing – review & editing. FN: Conceptualization, Methodology, Project administration, Resources, Writing – review & editing. DT: Conceptualization, Funding acquisition, Methodology, Project administration, Resources, Supervision, Writing – review & editing. AD: Investigation, Resources, Supervision, Writing – review & editing. SaM: Conceptualization, Data curation, Funding acquisition, Methodology, Project administration, Resources, Supervision, Validation, Writing – review & editing.

## Supplementary material

Supplementary Table 1 Excel file showing all transcripts that were differentially expressed (p<0.01, |log_2_ fold-change| > 0.1) and their equivalent HGNC IDs along with gene description where each excel sheet represent either up or downregulated DEGs for V vs. M comparison of gut regions/phase. BioMart shows gene biotype to be all protein-coding except for two (Ig V genes). Raw and (DESeq2) normalised counts for each sample for M and V fish (n=6 each/phase) are shown. The DESeq2-analysed output of SARTools is also presented, with DEGs ordered by the highest fold change.

Supplementary Table 2 Excel file showing in each sheet either activated or inhibited pathways/GO terms associated with up- or downregulated genes respectively. Gene set enrichment analysis was carried out in DAVID (at p<0.01 and minimum gene count of 3) and results displayed as GO:BP terms (**Figures 3**, **5**, **6**), KEGG (**Figure 4**), and reactome (**Supplementary Figure S1**) pathways for V vs. M comparison in whole intestine (at end of stimulus phase), middle or distal intestine (at end of challenge phase). Other GO terms results (GO:CC; Cellular Process and GO:MF; Metabolic Function) are also presented but is not the focus of this study.

Supplementary Table 3 Excel file showing upstream regulators predicted by IPA (at p<0.01, |z-score|> 2 where z-score>2 for activated regulators and z-score<-2 for inhibited regulators) that drives this study gene expression in V vs. M fish comparison in whole intestine (stimulus phase), middle or distal intestine (challenge phase). Each excel sheet represent either activated or inhibited upstream regulators for each comparison.

Supplementary Figure 1 Reactome pathway analysis of V fish vs. M fish in whole intestine at stimulus phase **(A)** and middle **(B)** and distal **(C)** intestines at challenge phase. KEGG pathways have been filtered to show DEGs involved within each pathway greater than 3 counts (represented by the size of the dot), and a p-value of less than 0.01. Colors represent −log10 (p-value), with red being the highest.

Supplementary Figure 2 Ingenuity Pathway Analysis (IPA) of V fish vs. M fish in whole intestine at stimulus phase. IPA canonical pathways have been filtered to show pathways at -log(p-value) threshold of greater than 1.3. Pathways are ordered by z-scores (largest to smallest). Colors represent z-score, with orange being the activated (positive z-score) and blue being inhibited (negative z-score).

Supplementary Figure 3 Ingenuity Pathway Analysis (IPA) of V fish vs. M fish in middle **(A)** and distal **(B)** intestines at challenge phase. IPA canonical pathways have been filtered to show pathways at -log(p-value) threshold of greater than 1.3. Pathways are ordered by z-scores (largest to smallest). Colors represent z-score, with orange being the activated (positive z-score) and blue being inhibited (negative z-score).

Additional file 1 Further feeding trial details including RAS conditions, formulation, proximate and fatty acid compositions, and Protocol of gut sampling.

Additional file 2 GO terms hierarchically organised by PANTHER tree-based classification tool. Main aim of this GO analysis is to categorise effects of NP on gene expression of V vs. M comparisons in whole intestine (stimulus phase), middle or distal intestine (challenge phase).
