# Supplementary figures and images for: Modulation of metabolic and immunoregulatory pathways in the gut transcriptome of Atlantic salmon (Salmo salar L.) after early nutritional programming during first feeding with plant-based diet
Source: Front Immunol. 2024 Jul 2;15:1412821. doi: 10.3389/fimmu.2024.1412821 (PMC11249740; doi:10.3389/fimmu.2024.1412821)

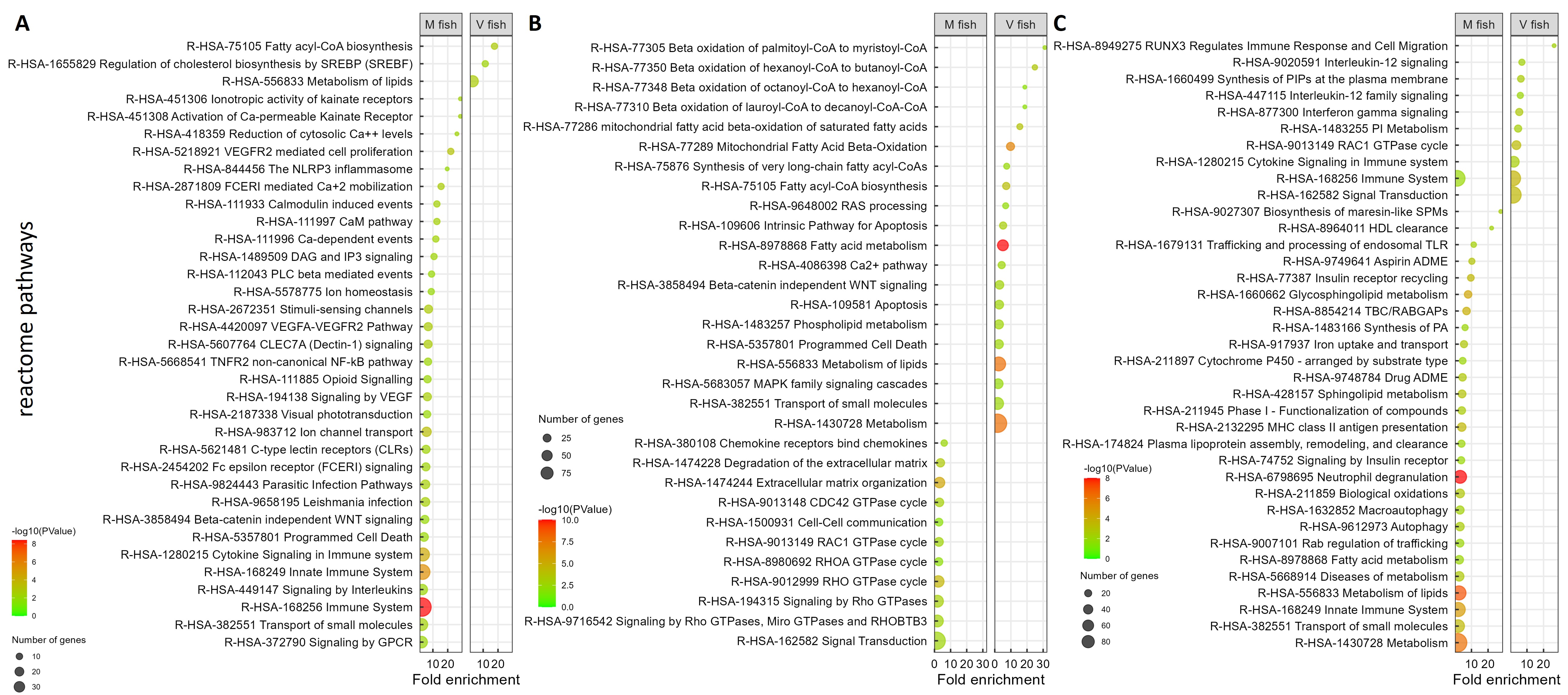

Supplement: Supplementary Figure 1 — Reactome pathway analysis of V fish vs. M fish in whole intestine at stimulus phase (A) and middle (B) and distal (C) intestines at challenge phase. KEGG pathways have been filtered to show DEGs involved within each pathway greater than 3 counts (represented by the size of the dot), and a p-value of less than 0.01. Colors represent −log10 (p-value), with red being the highest. [file Image_1.jpeg]

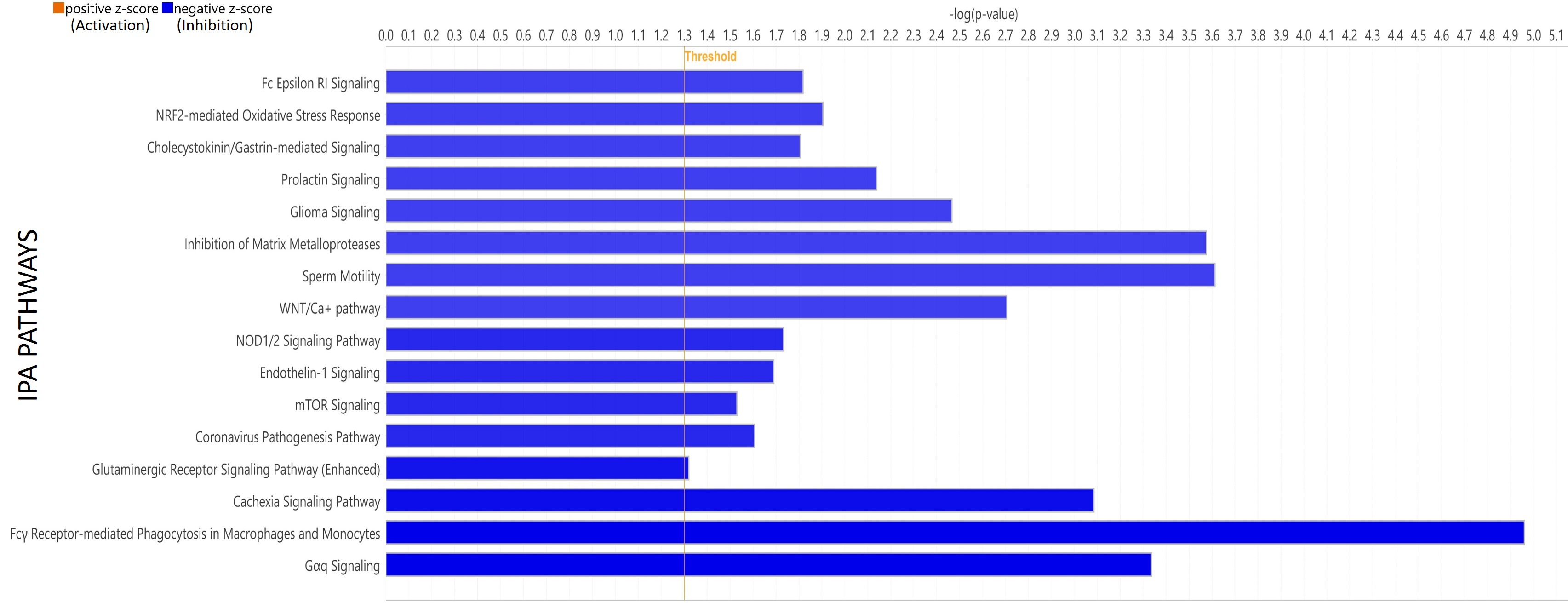

Supplement: Supplementary Figure 2 — Ingenuity Pathway Analysis (IPA) of V fish vs. M fish in whole intestine at stimulus phase. IPA canonical pathways have been filtered to show pathways at -log(p-value) threshold of greater than 1.3. Pathways are ordered by z-scores (largest to smallest). Colors represent z-score, with orange being the activated (positive z-score) and blue being inhibited (negative z-score). [file Image_2.jpeg]

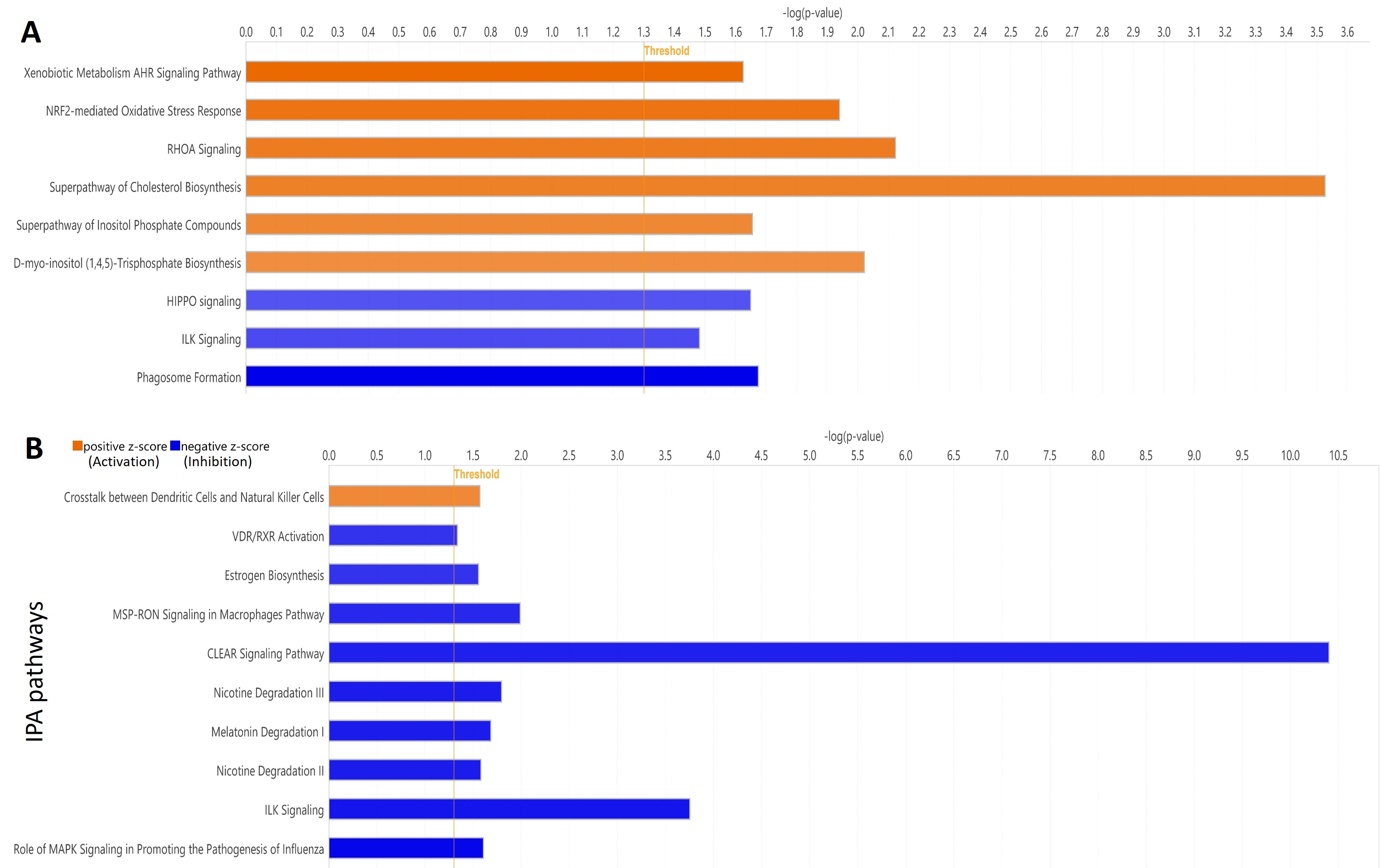

Supplement: Supplementary Figure 3 — Ingenuity Pathway Analysis (IPA) of V fish vs. M fish in middle (A) and distal (B) intestines at challenge phase. IPA canonical pathways have been filtered to show pathways at -log(p-value) threshold of greater than 1.3. Pathways are ordered by z-scores (largest to smallest). Colors represent z-score, with orange being the activated (positive z-score) and blue being inhibited (negative z-score). [file Image_3.jpeg]
