# Supplementary material for: Modulation of metabolic and immunoregulatory pathways in the gut transcriptome of Atlantic salmon (Salmo salar L.) after early nutritional programming during first feeding with plant-based diet
Source: Front Immunol. 2024 Jul 2;15:1412821. doi: 10.3389/fimmu.2024.1412821 (PMC11249740; doi:10.3389/fimmu.2024.1412821)

Additional details of the fish used in the trials.

On 20th December 2018, a total of 2,700 Atlantic salmon eggs were acquired from Mowi (Tveitevåg, Norway) where the spawning occurred at 388 degree-days (dd) post fertilisation (spawning date: 18th October 2018). Eggs were divided equally in 150 L tanks until they hatched (alevin stage) between 500 dd until 850 dd (i.e., by 6 January 2019, fry stage). Water temperature started at 7.0 ± 0.4 °C and maintained throughout egg incubation and alevin stage and gradually increased and kept at 13.1 ± 0.5 °C for the rest of the feeding trial while exposing fish throughout the trail to 24h artificial light. Survival to fry stage was 97.7 ± 0.6 %. Water volume was increased for fry stage to 300 L per tank. Water temperature, oxygen level (86 % saturation, 8-9 mg/L), pH (7.16 ± 0.2), nitrogen NO_2 (0.32 ± 0.2 mg/L), total ammonia nitrogen (0.13 ± 0.7 mg/L) and chloride (135 ± 13mg/L) were controlled. After a baseline sampling, 278 ± 2 fish remained per tank and feeding trial of NP experimental design was initiated on 21st February 2019 (875 dd). Fish were fed by automatic feeders (Arvo-tec TD2000; Huutokoski, Finland) with user interface (ArvoPRO) for 22 h/day, with a pause for 1 h at 09:00 and 16:00 for feed collection. Daily feeding rate was based on feeding tables (BioMar Inicio Plus), plus 10 % to ensure excess feed was provided to enable accurate estimates of feed consumed.

Formulation, proximate and fatty acid compositions of standard marine diets (M_S and M_I) and low fishmeal/fish oil vegetable-based diets (V_S and V_C) used in respective feeding phases.

**Experimental phase:**	**Stimulus**		**Intermediate**	**Challenge**
**Diet:**	**M_S**	**V_S**	**M_I**	**V_C**
**Ingredients (g/kg)**				
**Marine meals**				
Fishmeal*	667	0	490	50
Krill meal†	100	25	0	0
Fish peptones†	50	25	0	50
**Vegetable meals**				
SPC‡	0	155	161	90
Wheat products§	70	271	175	281
PPC‖	0	300	20	250
Other vegetable sources	30	20	0	60
**Fish oil****	44	0	86	0
**Rapeseed oil§**	0	67	51	141
**Lecithin¶**	5.2	36.5	5	5
**Vitamins and minerals††**	28.7	72.5	23.8	65.7
**Amino acids‡‡**	1.3	36.4	1.7	21.45
**Analysed proximate composition**				
Ash crude (%)	12.2	8.8	10.1	7.3
Lipid crude (%)	13.3	13.4	17.8	16.3
Protein crude (%)	60.0	56.0	52.6	51.8
Energy gross (MJ/kg)	20.6	21.1	21.8	22.3
**All fatty acids (% total fatty acids)**				
SFA	29.0	17.0	20.9	10.4
OA (18:1*n*-9)	38.7	12.8	26.3	52.6
MUFA	28.8	45.9	41.8	55.4
LA (18:2*n*-6)	5.7	21.5	10.3	23.6
ARA (20:4*n*-6)	0.1	0.7	0.5	0.1
ALA (18:3*n*-3)	1.8	7.0	4.2	8.9
EPA (20:5*n*-3)	11.9	3.4	8.1	0.5
DHA (22:6*n*-3)	14.1	2.5	8.2	0.7
PUFA	42.2	37.1	37.3	34.2
n-3 LC-PUFA	27.7	6.1	17.7	1.2

ALA, α-linolenic acid; ARA, arachidonic acid; LA, linoleic acid; LC-PUFA, long-chain polyunsaturated fatty acid; MUFA, monounsaturated fatty acid; OA, Oleic acid; PPC, pea protein concentrate; SFA, saturated fatty acid; SPC, soya protein concentrate.

* Feed Services Bremen, Bremen, Germany.
† Aker BioMarine, Lysaker, Norway.
‡ Caramuru, Itumbiara, Brazil.
§ Cargill, Minnesota, US.
‖ Agrident, Amsterdam, Netherlands.
** ED&F Man, London, UK.
¶ Nova Nutriway GmbH, Hamburg, Germany
†† DSM, Heerlen, Netherlands.
‡‡ Evonik, Essen, Germany.

Dissection of gut regions

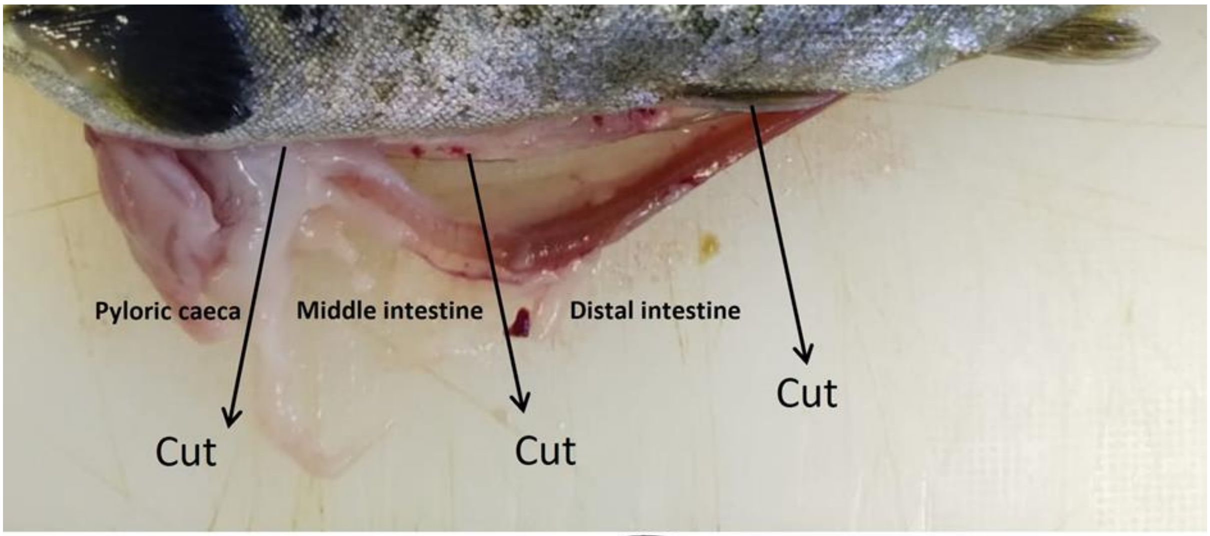

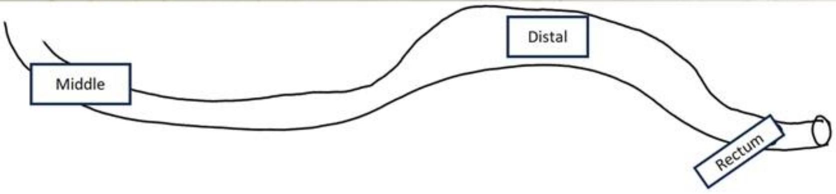

Supplement: Additional file 1 — Further feeding trial details including RAS conditions, formulation, proximate and fatty acid compositions, and Protocol of gut sampling. [file DataSheet_1.pdf]
