# Supplementary material for: Modulation of metabolic and immunoregulatory pathways in the gut transcriptome of Atlantic salmon (Salmo salar L.) after early nutritional programming during first feeding with plant-based diet
Source: Front Immunol. 2024 Jul 2;15:1412821. doi: 10.3389/fimmu.2024.1412821 (PMC11249740; doi:10.3389/fimmu.2024.1412821)
Supplement: Additional file 2 — GO terms hierarchically organised by PANTHER tree-based classification tool. Main aim of this GO analysis is to categorise effects of NP on gene expression of V vs. M comparisons in whole intestine (stimulus phase), middle or distal intestine (challenge phase). [file DataSheet_2.pdf]

PANTHER Classification System

The mission of the PANTHER knowledgebase is to support biomedical and other research by providing **comprehensive information about the evolution of protein-coding gene families**, particularly protein phylogeny, function and genetic variation impacting that function. Learn more

PANTHER will be unavailable on September 15th, 2023 for maintenance starting at 10:00AM PT for approximately 6 hours

search keyword All Go

Home About Data Version Tools API/Services Publications Workspace Downloads FAQ/Help/Tutorial Login Register Contact us

Current Release: PANTHER 17.0 | 15,619 family phylogenetic trees | 143 species | News
Whole genome function views

Analysis Summary: Please report in publication

Analysis Type: PANTHER Overrepresentation Test (Released 20230705)

Annotation Version and Release Date: GO Ontology database DOI: 10.5281/zenodo.7942786 Released 2023-05-10

Analyzed List: up_stim.txt (Homo sapiens)
There are duplicate IDs in the file. The unique set of IDs will be used. Change

Reference List: allgenes.txt (Homo sapiens)
There are duplicate IDs in the file. The unique set of IDs will be used. Change

Annotation Data Set: GO biological process complete

Test Type: (•) Fisher's Exact ( ) Binomial

Correction: (•) Calculate False Discovery Rate ( ) Use the Bonferroni correction for multiple testing ( ) No correction

Results

	Reference list	up_stim.txt
Uniquely Mapped IDS:	14222 out of 14281	77 out of 80
Unmapped IDs:	94	0
Multiple mapping information:	957	3

Export Table XML with user input ids JSON with user input ids

No statistically significant results. Click to see all results.

About | Release Information | Contact Us | System Requirements | Privacy Policy | Disclaimer

GENEONTOLOGY Unifying Biology

GLOBAL CORE BIODATA RESOURCE

© Copyright 2023 Paul Thomas All Rights Reserved.

PANTHER Classification System

The mission of the PANTHER knowledgebase is to support biomedical and other research by providing **comprehensive information about the evolution of protein-coding gene families**, particularly protein phylogeny, function and genetic variation impacting that function. Learn more

PANTHER will be unavailable on September 15th, 2023 for maintenance starting at 10:00AM PT for approximately 6 hours

search keyword All Go

Home About Data Version Tools API/Services Publications Workspace Downloads FAQ/Help/Tutorial Login Register Contact us

Current Release: PANTHER 17.0 | 15,619 family phylogenetic trees | 143 species | News
Whole genome function views

Analysis Summary: Please report in publication

Analysis Type: PANTHER Overrepresentation Test (Released 20230705)

Annotation Version and Release Date: GO Ontology database DOI: 10.5281/zenodo.7942786 Released 2023-05-10

Analyzed List: down_stim.txt (Homo sapiens)
There are duplicate IDs in the file. The unique set of IDs will be used. Change

Reference List: allgenes.txt (Homo sapiens)
There are duplicate IDs in the file. The unique set of IDs will be used. Change

Annotation Data Set: GO biological process complete

Test Type: Fisher's Exact Binomial

Correction: Calculate False Discovery Rate Use the Bonferroni correction for multiple testing No correction

Results

	Reference list	down_stim.txt
Uniquely Mapped IDS:	14222 out of 14281	119 out of 124
Unmapped IDs:	94	0
Multiple mapping information:	957	5

Export Table XML with user input ids JSON with user input ids

Displaying only results for FDR P < 0.05, click here to display all results

	allgenes.txt (REF)	down_stim.txt (Hierarchy				
GO biological process complete	#	#	expected	Fold Enrichment	+/-	rav
regulation of cardiac muscle contraction by regulation of the release of sequestered calcium ion	17	4	.15	27.10	+	2
↳intracellular signal transduction	1370	33	11.90	2.77	+	5
↳regulation of release of sequestered calcium ion into cytosol by sarcoplasmic reticulum	25	5	.22	23.03	+	5
↳regulation of release of sequestered calcium ion into cytosol	67	6	.58	10.31	+	3
↳regulation of sequestering of calcium ion	115	8	1.00	8.01	+	1
regulation of ryanodine-sensitive calcium-release channel activity	21	4	.18	21.94	+	5
innate immune response	484	15	4.20	3.57	+	2
↳defense response to other organism	633	18	5.50	3.27	+	1
↳response to other organism	915	22	7.94	2.77	+	1
↳biological process involved in interspecies interaction between organisms	1074	24	9.33	2.57	+	1
↳response to external biotic stimulus	918	22	7.97	2.76	+	1
↳response to biotic stimulus	953	23	8.27	2.78	+	8
↳defense response	955	25	8.29	3.01	+	7
↳immune response	939	25	8.15	3.07	+	5
↳immune system process	1477	33	12.82	2.57	+	3
regulation of immune response	640	18	5.56	3.24	+	1
↳regulation of immune system process	1122	24	9.74	2.46	+	3
positive regulation of immune system process	706	19	6.13	3.10	+	1
nucleic acid metabolic process	1997	3	17.34	.17	-	2

About | Release Information | Contact Us | System Requirements | Privacy Policy | Disclaimer

GLOBAL CORE BIODATA RESOURCE

© Copyright 2023 Paul Thomas All Rights Reserved.

PANTHER Classification System

The mission of the PANTHER knowledgebase is to support biomedical and other research by providing **comprehensive information about the evolution of protein-coding gene families**, particularly protein phylogeny, function and genetic variation impacting that function. Learn more

PANTHER will be unavailable on September 15th, 2023 for maintenance starting at 10:00AM PT for approximately 6 hours

search keyword All Go

Home About Data Version Tools API/Services Publications Workspace Downloads FAQ/Help/Tutorial Login Register Contact us

Current Release: PANTHER 17.0 | 15,619 family phylogenetic trees | 143 species | News
Whole genome function views

Analysis Summary: Please report in publication

Analysis Type: PANTHER Overrepresentation Test (Released 20230705)

Annotation Version and Release Date: GO Ontology database DOI: 10.5281/zenodo.7942786 Released 2023-05-10

Analyzed List: up_middle.txt (Homo sapiens)
There are duplicate IDs in the file. The unique set of IDs will be used. Change

Reference List: allgenes.txt (Homo sapiens)
There are duplicate IDs in the file. The unique set of IDs will be used. Change

Annotation Data Set: GO biological process complete

Test Type: (•) Fisher's Exact ( ) Binomial

Correction: (•) Calculate False Discovery Rate ( ) Use the Bonferroni correction for multiple testing ( ) No correction

Results

	Reference list	up_middle.txt
Uniquely Mapped IDS:	14222 out of 14281	410 out of 417
Unmapped IDs:	94	2
Multiple mapping information:	957	10

Export Table XML with user input ids JSON with user input ids

Displaying only results for FDR P < 0.05, click here to display all results

	allgenes.txt (REF)	up_middle.txt (Hierarchy NEW!)					
GO biological process complete	#	#	expected	Fold Enrichment	+/-	raw P value	FDR
fatty acid beta-oxidation	57	12	1.66	7.21	+	4.74E-07	5.15E-04
↳fatty acid catabolic process	82	16	2.39	6.68	+	1.49E-08	4.53E-05
↳cellular lipid catabolic process	168	19	4.91	3.87	+	1.57E-06	1.26E-03
↳cellular lipid metabolic process	758	56	22.13	2.53	+	6.30E-10	3.19E-06
↳lipid metabolic process	998	68	29.14	2.33	+	2.80E-10	4.26E-06
↳metabolic process	6766	248	197.56	1.26	+	1.27E-06	1.07E-03
↳cellular catabolic process	926	53	27.04	1.96	+	5.36E-06	3.26E-03
↳catabolic process	1662	84	48.53	1.73	+	1.02E-06	9.11E-04
↳lipid catabolic process	237	21	6.92	3.03	+	1.52E-05	7.70E-03
↳monocarboxylic acid catabolic process	104	16	3.04	5.27	+	2.76E-07	3.23E-04
↳carboxylic acid catabolic process	210	21	6.13	3.42	+	2.72E-06	1.80E-03
↳organic acid catabolic process	210	21	6.13	3.42	+	2.72E-06	1.88E-03

↳organic acid metabolic process	712	49	20.79	2.36	+	6.86E-08	1.04E-04
↳small molecule metabolic process	1410	85	41.17	2.06	+	3.22E-10	2.45E-06
↳small molecule catabolic process	300	24	8.76	2.74	+	1.84E-05	9.04E-03
↳carboxylic acid metabolic process	690	49	20.15	2.43	+	3.36E-08	7.30E-05
↳oxoacid metabolic process	709	49	20.70	2.37	+	6.16E-08	1.04E-04
↳monocarboxylic acid metabolic process	417	35	12.18	2.87	+	7.47E-08	1.03E-04
↳fatty acid metabolic process	262	30	7.65	3.92	+	1.11E-09	4.23E-06
↳fatty acid oxidation	74	13	2.16	6.02	+	9.74E-07	9.25E-04
↳lipid oxidation	79	13	2.31	5.64	+	1.87E-06	1.35E-03
↳lipid modification	153	17	4.47	3.81	+	6.90E-06	4.03E-03
endosome to lysosome transport	68	10	1.99	5.04	+	6.82E-05	2.66E-02
↳transport	3202	143	93.50	1.53	+	5.60E-08	1.06E-04
↳establishment of localization	3347	147	97.73	1.50	+	9.00E-08	1.14E-04
↳localization	3850	166	112.42	1.48	+	2.07E-08	5.24E-05
↳intracellular transport	1217	67	35.54	1.89	+	9.39E-07	9.52E-04
endosomal transport	216	20	6.31	3.17	+	1.34E-05	7.05E-03
organic anion transport	300	24	8.76	2.74	+	1.84E-05	8.75E-03
↳organic substance transport	1883	91	54.98	1.66	+	1.81E-06	1.38E-03
autophagy	285	22	8.32	2.64	+	6.73E-05	2.76E-02
↳process utilizing autophagic mechanism	285	22	8.32	2.64	+	6.73E-05	2.69E-02
purine nucleotide metabolic process	359	26	10.48	2.48	+	5.43E-05	2.29E-02
↳organophosphate metabolic process	784	46	22.89	2.01	+	1.26E-05	6.82E-03
↳purine-containing compound metabolic process	385	26	11.24	2.31	+	1.27E-04	4.69E-02
↳organonitrogen compound metabolic process	4120	160	120.30	1.33	+	3.91E-05	1.75E-02
lipid biosynthetic process	517	36	15.10	2.38	+	4.73E-06	3.00E-03
membrane organization	656	39	19.15	2.04	+	5.11E-05	2.22E-02
nitrogen compound transport	1417	71	41.38	1.72	+	1.07E-05	6.02E-03
mRNA metabolic process	551	2	16.09	.12	-	3.40E-05	1.57E-02
↳RNA metabolic process	1450	20	42.34	.47	-	1.26E-04	4.80E-02

About | Release Information | Contact Us | System Requirements | Privacy Policy | Disclaimer

GENEONTOLOGY Unifying Biology GLOBAL CORE BIODATA RESOURCE

© Copyright 2023 Paul Thomas All Rights Reserved.

PANTHER Classification System

The mission of the PANTHER knowledgebase is to support biomedical and other research by providing **comprehensive information about the evolution of protein-coding gene families**, particularly protein phylogeny, function and genetic variation impacting that function. Learn more

PANTHER will be unavailable on September 15th, 2023 for maintenance starting at 10:00AM PT for approximately 6 hours

search keyword All Go

Home About Data Version Tools API/Services Publications Workspace Downloads FAQ/Help/Tutorial Login Register Contact us

Current Release: PANTHER 17.0 | 15,619 family phylogenetic trees | 143 species | News
Whole genome function views

Analysis Summary: Please report in publication

Analysis Type: PANTHER Overrepresentation Test (Released 20230705)

Annotation Version and Release Date: GO Ontology database DOI: 10.5281/zenodo.7942786 Released 2023-05-10

Analyzed List: down_middle.txt (Homo sapiens)
There are duplicate IDs in the file. The unique set of IDs will be used. Change

Reference List: allgenes.txt (Homo sapiens)
There are duplicate IDs in the file. The unique set of IDs will be used. Change

Annotation Data Set: GO biological process complete

Test Type: Fisher's Exact Binomial

Correction: Calculate False Discovery Rate Use the Bonferroni correction for multiple testing No correction

Results

	Reference list	down_middle.txt
Uniquely Mapped IDS:	14222 out of 14281	389 out of 394
Unmapped IDs:	94	3
Multiple mapping information:	957	5

Export Table XML with user input ids JSON with user input ids

Displaying only results for FDR P < 0.05, click here to display all results

	allgenes.txt (REF)
GO biological process complete	#
acute inflammatory response	57
↳inflammatory response	411
↳response to stimulus	6117
immunoglobulin mediated immune response	99
↳B cell mediated immunity	102
↳adaptive immune response based on somatic recombination of immune receptors built from immunoglobulin superfamily domains	160
↳adaptive immune response	298
↳immune response	939
↳immune system process	1477
↳lymphocyte mediated immunity	143
↳leukocyte mediated immunity	184
↳immune effector process	302
cell-matrix adhesion	119
↳cell-substrate adhesion	166
↳cell adhesion	774
positive regulation of T cell activation	180
↳positive regulation of leukocyte cell-cell adhesion	200
↳regulation of leukocyte cell-cell adhesion	273
↳regulation of cell-cell adhesion	364
↳regulation of cell adhesion	635

↳positive regulation of cell-cell adhesion	238
↳positive regulation of cell adhesion	383
↳positive regulation of cellular process	4615
↳positive regulation of biological process	5138
↳positive regulation of lymphocyte activation	243
↳positive regulation of leukocyte activation	277
↳positive regulation of immune system process	706
↳regulation of immune system process	1122
↳positive regulation of cell activation	288
↳regulation of cell activation	485
↳regulation of multicellular organismal process	2511
↳positive regulation of multicellular organismal process	1361
↳regulation of leukocyte activation	444
↳regulation of lymphocyte activation	374
↳regulation of T cell activation	274
humoral immune response	133
cartilage development	160
↳anatomical structure development	4343
↳developmental process	4715
↳connective tissue development	213
↳skeletal system development	461
cell chemotaxis	162
↳response to external stimulus	1855
↳cell migration	777
↳cell motility	904
T cell differentiation	157
↳T cell activation	243
↳lymphocyte activation	370
↳leukocyte activation	455
↳lymphocyte differentiation	243
↳mononuclear cell differentiation	276
↳leukocyte differentiation	353
↳cell differentiation	2968
↳cellular developmental process	2991
positive regulation of supramolecular fiber organization	157
regulation of lymphocyte differentiation	170
↳regulation of leukocyte differentiation	253
↳regulation of hemopoiesis	326
↳regulation of multicellular organismal development	1223
↳regulation of developmental process	2151
↳regulation of cell differentiation	1350
immune response-regulating cell surface receptor signaling pathway	182
↳signal transduction	3556
↳signaling	3820
↳cell communication	3958
↳regulation of immune response	640
↳regulation of response to stimulus	3288
↳cell surface receptor signaling pathway	1644
immune response-activating signaling pathway	224
↳activation of immune response	265
↳positive regulation of immune response	442

↳positive regulation of response to stimulus	1789
extracellular matrix organization	238
↳extracellular structure organization	239
↳external encapsulating structure organization	240
angiogenesis	301
↳anatomical structure morphogenesis	1995
↳blood vessel morphogenesis	384
↳tube morphogenesis	622
↳vasculature development	486
cellular response to growth factor stimulus	435
↳response to growth factor	465
positive regulation of cell differentiation	750
↳positive regulation of developmental process	1157
regulation of intracellular signal transduction	1507
↳regulation of signal transduction	2558
↳regulation of cell communication	2948
↳regulation of signaling	2940
regulation of cell population proliferation	1388

About | Release Information | Contact Us | System Requirements | Privacy Policy | Disclaimer GENEONTOLOGY Unifying Biology GLOBAL CORE BIODATA RESOURCE
© Copyright 2023 Paul Thomas All Rights Reserved.

PANTHER Classification System

The mission of the PANTHER knowledgebase is to support biomedical and other research by providing **comprehensive information about the evolution of protein-coding gene families**, particularly protein phylogeny, function and genetic variation impacting that function. Learn more

PANTHER will be unavailable on September 15th, 2023 for maintenance starting at 10:00AM PT for approximately 6 hours

search keyword All Go

Home About Data Version Tools API/Services Publications Workspace Downloads FAQ/Help/Tutorial Login Register Contact us

Current Release: PANTHER 17.0 | 15,619 family phylogenetic trees | 143 species | News
Whole genome function views

Analysis Summary: Please report in publication

Analysis Type: PANTHER Overrepresentation Test (Released 20230705)

Annotation Version and Release Date: GO Ontology database DOI: 10.5281/zenodo.7942786 Released 2023-05-10

Analyzed List: up_distal.txt (Homo sapiens)
! There are duplicate IDs in the file. The unique set of IDs will be used. Change

Reference List: allgenes.txt (Homo sapiens)
! There are duplicate IDs in the file. The unique set of IDs will be used. Change

Annotation Data Set: GO biological process complete

Test Type: (●) Fisher's Exact ( ) Binomial

Correction: (●) Calculate False Discovery Rate ( ) Use the Bonferroni correction for multiple testing ( ) No correction

Results

	Reference list	up_distal.txt
Uniquely Mapped IDS:	14222 out of 14281	260 out of 267
Unmapped IDs:	94	1
Multiple mapping information:	957	8

Export Table XML with user input ids JSON with user input ids

Displaying only results for FDR P < 0.05, click here to display all results

allgene

GO biological process complete
positive regulation of CD8-positive, alpha-beta T cell differentiation
↳positive regulation of CD8-positive, alpha-beta T cell activation
↳positive regulation of alpha-beta T cell activation
↳positive regulation of T cell activation
↳positive regulation of leukocyte cell-cell adhesion
↳regulation of leukocyte cell-cell adhesion
↳regulation of cell-cell adhesion
↳regulation of cellular process
↳positive regulation of cell-cell adhesion
↳positive regulation of cell adhesion
↳positive regulation of lymphocyte activation
↳positive regulation of leukocyte activation
↳positive regulation of immune system process
↳regulation of immune system process
↳positive regulation of cell activation
↳regulation of cell activation
↳regulation of multicellular organismal process
↳positive regulation of multicellular organismal process
↳regulation of leukocyte activation
↳regulation of lymphocyte activation

↳regulation of T cell activation
↳regulation of alpha-beta T cell activation
↳regulation of T cell differentiation
↳regulation of lymphocyte differentiation
↳regulation of leukocyte differentiation
↳regulation of hemopoiesis
↳positive regulation of lymphocyte differentiation
↳positive regulation of leukocyte differentiation
↳positive regulation of hemopoiesis
positive regulation of NK T cell proliferation
↳positive regulation of NK T cell activation
↳regulation of alpha-beta T cell proliferation
↳regulation of T cell proliferation
↳regulation of lymphocyte proliferation
↳regulation of mononuclear cell proliferation
↳regulation of leukocyte proliferation
↳positive regulation of alpha-beta T cell proliferation
↳positive regulation of T cell proliferation
↳positive regulation of lymphocyte proliferation
↳positive regulation of mononuclear cell proliferation
↳positive regulation of leukocyte proliferation
positive regulation of natural killer cell proliferation
↳regulation of natural killer cell proliferation
↳regulation of natural killer cell activation
↳positive regulation of natural killer cell activation
T cell migration
↳lymphocyte migration
↳cell migration
↳immune system process
leukocyte cell-cell adhesion
regulation of CD4-positive, alpha-beta T cell differentiation
T-helper cell differentiation
↳CD4-positive, alpha-beta T cell differentiation involved in immune response
↳alpha-beta T cell differentiation involved in immune response
↳T cell differentiation involved in immune response
↳T cell differentiation
↳T cell activation
↳lymphocyte activation
↳leukocyte activation
↳cell activation
↳lymphocyte differentiation
↳mononuclear cell differentiation
↳leukocyte differentiation
↳hemopoiesis
↳T cell activation involved in immune response
↳lymphocyte activation involved in immune response
↳leukocyte activation involved in immune response
↳cell activation involved in immune response
↳immune effector process
↳immune response
↳response to stimulus

↳alpha-beta T cell activation involved in immune response
↳alpha-beta T cell activation
↳alpha-beta T cell differentiation
↳CD4-positive, alpha-beta T cell differentiation
↳CD4-positive, alpha-beta T cell activation
B cell receptor signaling pathway
↳signal transduction [illegible]
↳cellular response to stimulus [illegible]
↳signaling [illegible]
↳cell communication [illegible]
↳regulation of immune response [illegible]
regulation of T cell mediated immunity
↳regulation of adaptive immune response based on somatic recombination of immune receptors built from immunoglobulin superfamily domains [illegible]
positive regulation of tumor necrosis factor production
↳regulation of cytokine production [illegible]
↳positive regulation of tumor necrosis factor superfamily cytokine production
↳positive regulation of cytokine production [illegible]
↳positive regulation of gene expression [illegible]
B cell differentiation [illegible]
myeloid cell differentiation [illegible]
apoptotic signaling pathway [illegible]
response to other organism [illegible]
↳biological process involved in interspecies interaction between organisms [illegible]
↳response to external biotic stimulus [illegible]
↳response to biotic stimulus [illegible]
↳response to external stimulus [illegible]
phosphorylation [illegible]
regulation of hydrolase activity [illegible]
intracellular signal transduction [illegible]
positive regulation of molecular function [illegible]

About | Release Information | Contact Us | System Requirements | Privacy Policy | Disclaimer

© Copyright 2023 Paul Thomas All Rights Reserved.

PANTHER Classification System

The mission of the PANTHER knowledgebase is to support biomedical and other research by providing **comprehensive information about the evolution of protein-coding gene families**, particularly protein phylogeny, function and genetic variation impacting that function. Learn more

PANTHER will be unavailable on September 15th, 2023 for maintenance starting at 10:00AM PT for approximately 6 hours

search keyword All Go

Home About Data Version Tools API/Services Publications Workspace Downloads FAQ/Help/Tutorial Login Register Contact us

Current Release: PANTHER 17.0 | 15,619 family phylogenetic trees | 143 species | News
Whole genome function views

Analysis Summary: Please report in publication

Analysis Type: PANTHER Overrepresentation Test (Released 20230705)

Annotation Version and Release Date: GO Ontology database DOI: 10.5281/zenodo.7942786 Released 2023-05-10

Analyzed List: down_distal.txt (Homo sapiens)
There are duplicate IDs in the file. The unique set of IDs will be used. Change

Reference List: allgenes.txt (Homo sapiens)
There are duplicate IDs in the file. The unique set of IDs will be used. Change

Annotation Data Set: GO biological process complete

Test Type: (•) Fisher's Exact ( ) Binomial

Correction: Calculate False Discovery Rate Use the Bonferroni correction for multiple testing No correction

Results

	Reference list	down_distal.txt
Uniquely Mapped IDS:	14222 out of 14281	347 out of 361
Unmapped IDs:	94	1
Multiple mapping information:	957	12

Export Table XML with user input ids JSON with user input ids

Displaying only results for FDR P < 0.05, click here to display all results

	allgenes.txt (REF)	down_distal.txt (▼ Hierarch			
GO biological process complete	#	#	expected	Fold Enrichment	+/-
xenobiotic detoxification by transmembrane export across the plasma membrane	9	5	.23	21.98	+
↳xenobiotic export from cell	12	5	.30	16.48	+
glycolipid catabolic process	13	6	.33	18.26	+
↳membrane lipid metabolic process	179	19	4.52	4.20	+
↳cellular lipid metabolic process	758	60	19.16	3.13	+
↳lipid metabolic process	998	69	25.23	2.74	+
↳metabolic process	6766	211	171.03	1.23	+
↳organic substance catabolic process	1428	76	36.10	2.11	+
↳catabolic process	1662	89	42.01	2.12	+
↳membrane lipid catabolic process	31	9	.78	11.49	+
↳cellular lipid catabolic process	168	19	4.25	4.47	+
↳cellular catabolic process	926	59	23.41	2.52	+
↳lipid catabolic process	237	25	5.99	4.17	+
ceramide catabolic process	17	6	.43	13.96	+
↳ceramide metabolic process	85	10	2.15	4.65	+
↳sphingolipid metabolic process	138	17	3.49	4.87	+
↳sphingolipid catabolic process	27	7	.68	10.26	+
↳organonitrogen compound catabolic process	961	48	24.29	1.98	+
antigen processing and presentation of exogenous peptide antigen via MHC class II	19	6	.48	12.49	+
↳antigen processing and presentation of exogenous peptide antigen	26	6	.66	9.13	+

↳antigen processing and presentation of peptide antigen via MHC class II	21	6	.53	11.30	+
↳antigen processing and presentation of peptide or polysaccharide antigen via MHC class II	22	6	.56	10.79	+
lysosomal lumen acidification	21	6	.53	11.30	+
↳vacuolar acidification	29	9	.73	12.28	+
↳intracellular pH reduction	45	9	1.14	7.91	+
↳regulation of intracellular pH	76	10	1.92	5.21	+
↳regulation of cellular pH	80	10	2.02	4.94	+
↳regulation of pH	88	11	2.22	4.94	+
↳chemical homeostasis	744	39	18.81	2.07	+
↳regulation of lysosomal lumen pH	29	7	.73	9.55	+
↳vacuole organization	168	15	4.25	3.53	+
estrogen metabolic process	23	6	.58	10.32	+
↳steroid metabolic process	205	17	5.18	3.28	+
vitamin transport	37	7	.94	7.48	+
glycosphingolipid metabolic process	56	8	1.42	5.65	+
fatty acid biosynthetic process	96	13	2.43	5.36	+
↳monocarboxylic acid biosynthetic process	140	14	3.54	3.96	+
↳carboxylic acid biosynthetic process	222	17	5.61	3.03	+
↳carboxylic acid metabolic process	690	48	17.44	2.75	+
↳oxoacid metabolic process	709	49	17.92	2.73	+
↳organic acid metabolic process	712	49	18.00	2.72	+
↳small molecule metabolic process	1410	76	35.64	2.13	+
↳organic acid biosynthetic process	223	17	5.64	3.02	+
↳small molecule biosynthetic process	388	27	9.81	2.75	+
↳monocarboxylic acid metabolic process	417	39	10.54	3.70	+
↳fatty acid metabolic process	262	26	6.62	3.93	+
↳lipid biosynthetic process	517	33	13.07	2.53	+
hexose metabolic process	146	16	3.69	4.34	+
↳monosaccharide metabolic process	172	17	4.35	3.91	+
↳carbohydrate metabolic process	385	25	9.73	2.57	+
cellular response to nutrient levels	208	16	5.26	3.04	+

About | Release Information | Contact Us | System Requirements | Privacy Policy | Disclaimer

© Copyright 2023 Paul Thomas All Rights Reserved.
